# RTEL1 mutation modifies dyskeratosis congenita caused by a telomerase RNA template mutation

**DOI:** 10.1038/s41467-026-76650-w

**Published:** 2026-08-26

**Authors:** Nathaniel Deimler, Alex Orioli, Laura Holthöfer, Matthias Linke, Nadine Bobon, Verena Engelhardt, Lars Erichsen, Norbert W. Paul, Robert Meyer, Fabian Beier, Susann Schweiger, Peter Baumann

**Affiliations:** 1 Department of Biology, Hanns-Dieter-Hüsch-Weg 15, Johannes Gutenberg University, Mainz, Germany; 2 Institute for Quantitative and Computational Biosciences, Johannes von Müller Weg 6, Mainz, Germany; 3 Institute of Human Genetics, University Medical Center of the Johannes Gutenberg University Mainz, Langenbeckstr. 1, Mainz, Germany; 4 Institute for the History, Philosophy, and Ethics of Medicine, Johannes Gutenberg University Medical Center, Mainz, Germany; 5 Center for Integrated Oncology Aachen Bonn Cologne Düsseldorf (CIO ABCD), Aachen, Germany; 6 Center for Human Genetics and Genomic Medicine, Medical Faculty, RWTH Aachen University, Aachen, Germany; 7 Department of Hematology, Oncology, Hemostaseology, Stem Cell Transplantation, Medical Faculty, RWTH Aachen University, Aachen, Germany; 8 Institute for Molecular Biology, Ackermannweg 4, Mainz, Germany; 9 Leibniz Institute for Resilience Research, Wallstr. 7, Mainz, Germany; 10 Present Address: Laboratory of Genome Integrity, National Cancer Institute, NIH, Bethesda, MD USA

**Keywords:** Genetics research, Telomeres, Genomic analysis

## Abstract

Vertebrate telomeres, the sequences protecting the end of linear chromosomes, are composed of conserved hexameric GGTTAG repeats. Here we present a C50>A telomerase RNA template mutation that results in the incorporation of the variant telomeric repeat GTTTAG in a family with dyskeratosis congenita primarily presenting as idiopathic pulmonary fibrosis. The mutant telomerase shows reduced processivity, as demonstrated in direct activity assays and corroborated in vivo by Illumina whole-genome sequencing and Oxford Nanopore long-read telomere sequencing. The latter provides positional mutant repeat information that reveals the inheritance and maintenance of proximal mutant repeats in individuals who did not inherit the template mutation. Additionally, we describe an *RTEL1* nonsense mutation that is associated with very short telomeres in progeny harboring wild-type telomerase and inherited mutant telomeric repeats in the absence of overt clinical phenotypes. In contrast, co-inheritance of the *RTEL1* nonsense and *TERC r.C50*>*A* mutations coincide with severe early-onset DC phenotypes in two individuals.

## Abstract (web summary)

Telomere repeats are highly conserved, and disruptions in their maintenance can cause telomere disorders. Here, the authors identify a telomerase RNA mutation that generates variant telomeric repeats, reducing telomerase processivity and contributing to dyskeratosis congenita, with disease severity further influenced by RTEL1 variation.

## Introduction

Telomeres are nucleoprotein structures that constitute the terminal regions of eukaryotic chromosomes. In humans, telomeric DNA amounts to ~0.01% of the genome and caps each chromosome arm with 5–15 kb of GGTTAG repeats that terminate in a 100 to 200 nucleotide single-stranded G-rich 3’ overhang^1,2^. During cell proliferation, ~50–100 bp of terminal telomeric sequence is lost with each cell cycle due to the “end-replication problem”^3,4^. In the absence of compensatory mechanisms, this results in the progressive shortening of telomeres and ultimately leads to cellular senescence when telomeres reach a critical length^5^.

The ribonucleoprotein (RNP) complex telomerase counteracts telomere shortening in stem cells, many populations of transiently dividing cells, and the majority of cancer cells^1^. At its core, telomerase is comprised of the catalytic protein, Telomerase Reverse Transcriptase (TERT), and the non-coding Telomerase RNA (TR). Several other factors are necessary for telomerase biogenesis and function, including the dyskerin complex (DKC1, GAR1, NOP10, and NHP2) and TCAB1^6^, as well as multiple factors involved in TR maturation, such as the nuclear exosome complex, associated adapter complexes, and the nucleases PARN and TOE1^7,8^.

The telomerase RNA subunit TR contains an 11-nucleotide template region, five of which serve as an alignment region for the telomeric 3’ overhang, while the remaining six are reverse transcribed by TERT^9^. Once engaged with a telomere, human telomerase repeatedly adds telomeric repeats in an alignment, extension, and translocation cycle^1^.

Mutations in telomerase subunits and many genes involved in telomerase biogenesis and recruitment are associated with a group of premature-aging diseases, including dyskeratosis congenita (DC)^10^. While DC primarily manifests in a diagnostic triad of nail dysplasia, reticulate skin pigmentation, and leukoplakia, other symptoms such as Idiopathic Pulmonary Fibrosis (IPF), aplastic anemia, and liver disease are not uncommon^11^. Patients also have an increased risk of developing solid tumors and progressive bone marrow failure^11^. Related pathologies such as Hoyeraal-Hreidarsson syndrome and Revesz syndrome present with a similar set of clinical manifestations in addition to growth retardation, microcephaly, and intracranial calcifications^11^. Collectively, these phenotypically heterogeneous genetic disorders are referred to as Telomere Biology Disorders (TBDs)^12^. TBDs sensitize multiple tissues, including liver and lung, to environmental stress factors such as smoking, a high-fat diet, or elevated alcohol consumption^13,14^.

The molecular change underlying all telomere biology disorders is premature and accelerated telomere shortening. Telomere length measured in peripheral blood lymphocytes by fluorescence in situ hybridization in combination with flow cytometry (flow-FISH) has therefore emerged as the key diagnostic indicator of TBDs^15^. Adult-onset of TBD-associated clinical symptoms often correlates with age-adjusted telomere lengths below the tenth percentile, while pediatric-onset presentations of TBDs are commonly associated with telomere lengths below the first percentile^12^. Genetically, TBDs were first associated with mutations in the *DKC1* and *TERC* genes^16,17^, however, mutations in at least 17 genes involved in telomere maintenance have now been recognized as causative^12^. Most characterized mutations impact telomerase RNA maturation or stability, or diminish telomerase activity or recruitment to telomeres. The common outcome is insufficient replenishment of telomeric DNA and thus accelerated telomere shortening, eventually limiting the proliferative capacity of dividing cells.

Mutations in the template region of the telomerase RNA represent a distinct class, as reverse transcription of the altered template incorporates non-canonical repeats at chromosome ends, which can perturb telomere structure and impair binding of telomere-associated proteins. Unless shelterin binds the new sequence with sufficiently high affinity to retain its protective and regulatory functions, telomere length regulation and chromosome end protection will be compromised by template mutations. The requirement for telomere repeat sequences to co-evolve with the DNA binding specificity of several proteins, including TRF1, TRF2, and POT1, explains why telomeric sequences are slow to change across evolutionary time scales. In fact, the telomeric repeat sequence GGTTAG appears to be invariant across 400 million years of vertebrate evolution^18^.

Here, we describe the characterization of the template mutation C50>A in human TR (hTR) identified in a patient and three additional family members diagnosed with DC and IPF, respectively. While this work was in preparation, the same mutation was also described in an apparently unrelated family^19^. Our results are consistent with the conclusion by Hinchie et al. that the mutation affects telomere biology predominantly by reducing telomerase processivity. We further show here that while the ability of mutant hTR to bind a wild-type telomeric substrate was unaffected, the elongation of a mutant terminal repeat by wild-type telomerase was impaired. We expand on previous findings^19^ through the analysis of Illumina whole genome sequencing (WGS) and Oxford Nanopore Technologies (ONT) long read sequencing of the telomeres from several individuals carrying the mutation and their descendants. Unlike the previously described case, the burden of mutant telomeric repeats remained similar from one generation of heterozygous carrier to the next in the family examined here. Surprisingly, descendants of C50>A carriers, who themselves have only inherited wild-type *TERC*, carried a large burden of mutant telomeric repeats, suggesting minimal telomere turnover occurring from generation to generation. WGS analysis of a relative with short telomeres and no mutation in *TERC* led to the unexpected discovery of a second telomere maintenance-related mutation in the same family, a Q1178x nonsense allele of *RTEL1*, Regulator of Telomere Elongation Helicase 1, a protein responsible for unwinding G-quadruplexes and disassembling T-loops during DNA replication^20,21^. The combined effect of heterozygous mutations in two different genes involved in distinct telomere maintenance processes is likely to contribute to the severe pathologies observed in the index patient and his deceased twin sister.

## Results

### C50>A hTR mutation results in DC-like phenotypes

**Fig. 1:**
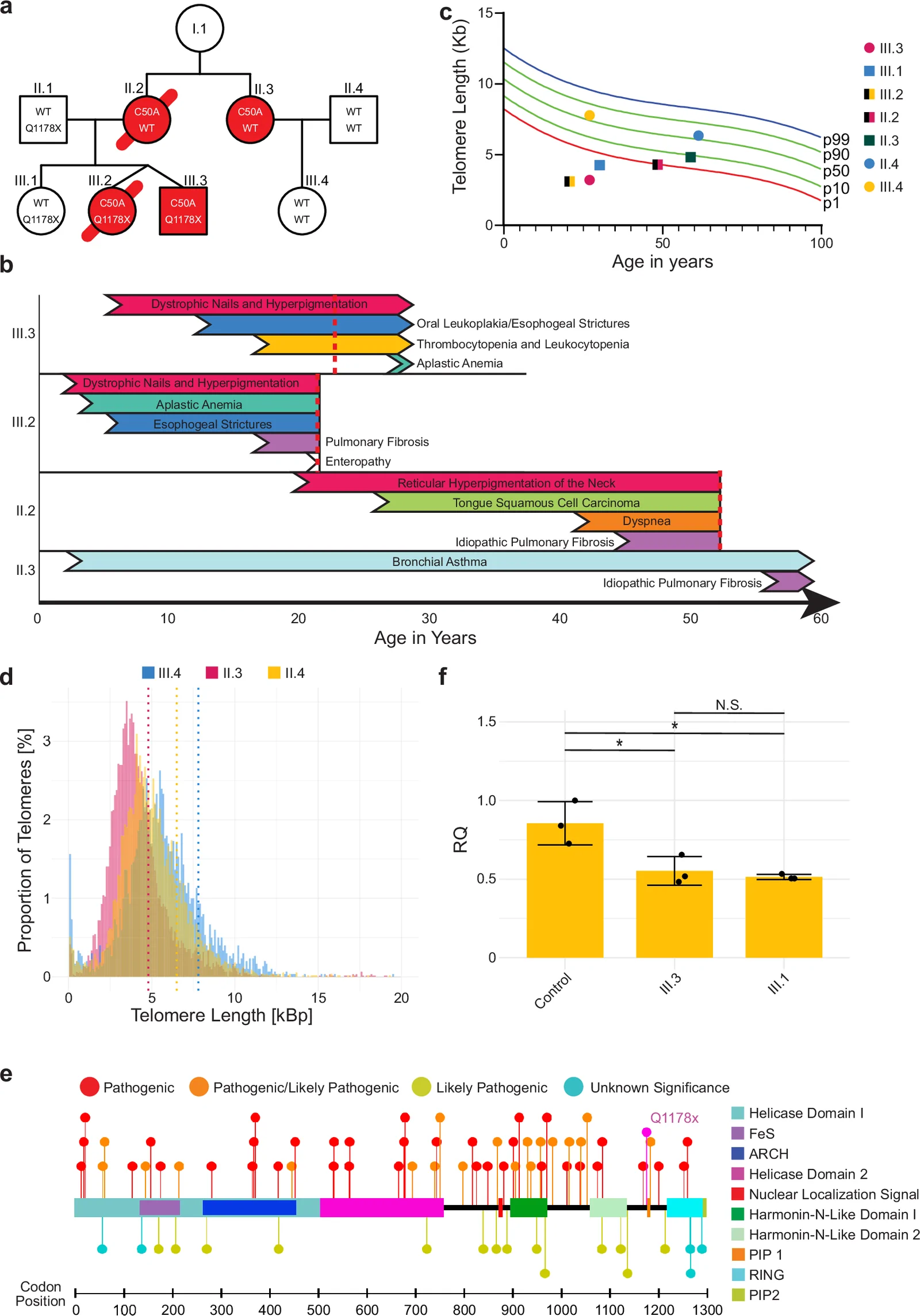
*r*.C50>A TERC mutation is associated with abnormally short telomeres and dyskeratosis congenita. **a** Genealogical tree of the proband and his family members annotated with the C50>A mutation or Q1178x RTEL1 mutation if the individual is heterozygous and wild-type if the individual is homozygous wild-type. Open circles and squares represent non-diagnosed females and males. Red circles and squares represent females and males diagnosed with dyskeratosis congenita or symptoms thereof. A solid red line indicates the individual is deceased. **b** Clinical timeline of the family members carrying the C50>A hTR mutation. **c** Lymphocyte flow-FISH data of all family members. Telomere length (kilobases, kb) is plotted against age (years). Curves represent the 1st (p1, red), 10th (p10, light green), 50th (p50, median, green), 90th (p90, dark green), and 99th (p99, blue) percentiles of telomere length across a population. **d** Telomere length distributions from Nanopore sequencing of II.3, II.4, and III.4. **e** Schematic illustration to scale of the protein formed from transcript RTEL1-203 containing relevant domains as described^58^. The nonsense mutation described here (Q1178x) is indicated by a purple circle, while other circles represent all nonsense mutations documented on ClinVar with at least one star. **f** qPCR analysis showing the expression levels of *RTEL1* mRNA isolated from cultivated fibroblasts of III.1, III3, and a healthy control individual. Bar height represents the mean of the biological replicates. Error bars denote ± 1 standard deviation (SD) centered on the mean (* = p.val < 0.05; two-sided *t* test; Control vs. III.1, p.val = 0.01; Control vs III.3, p.val = 0.03).

We describe a family carrying a C50>A mutation in the hTR template region that is associated with dyskeratosis congenita (DC)-like phenotypes. The index patient, subsequently referred to as individual III.3, and his twin sister, III.2 (Fig. 1a), were born after an uneventful twin pregnancy. III.3 appeared healthy until the age of 5, when he developed dystrophic nails and hyperpigmentation on the shoulders and neck. At age 12, he presented with oral leukoplakia requiring laser treatment and esophageal strictures that necessitated two rounds of dilation (Fig. 1b). Following the onset of severe fatigue at age 16, hematologic workup revealed thrombocytopenia and leukocytopenia. Around the same time, III.2 was diagnosed with DC-associated pulmonary fibrosis, with histopathology revealing severe alveolitis, bronchiolitis obliterans, and fibrosis. A diagnosis of dyskeratosis congenita was made at age 17, coinciding with the identification of the *TERC* variant *r.C50*>*A* by Sanger sequencing in III.3, later confirmed by whole genome sequencing (Supplementary Table 1) and deemed pathogenic based on its location within the template region of hTR. Ribo-depleted RNA sequencing from peripheral blood demonstrated that the mutant allele was expressed at similar levels to the wild-type allele, supporting the absence of an allelic expression bias or selective degradation of the mutant hTR (Supplementary Table 1). At age 26, III.3 was diagnosed with mild bone marrow failure. At the time of manuscript preparation, the now 28-year-old patient showed no signs of pulmonary fibrosis by computerized axial tomography and no other pulmonary dysfunctions were reported by the attending physicians.

In contrast, III.2 exhibited an earlier onset and increased disease severity compared to III.3, displaying the characteristic signs of DC early in life, including nail dystrophy and reticular hyperpigmentation. She was diagnosed with severe aplastic anemia at the age of 3 (Fig. 1b). She received two hematopoietic stem cell transplants at age 4: the first, from an unrelated donor, was rejected, while the second, from her father, successfully reconstituted her hematopoietic system. This is noteworthy, as the father’s cell harbored a - at the time of transplantation - undiagnosed mutation in *RTEL1* (see below). From age 5, she required multiple esophageal dilations for recurrent strictures and developed progressively worsening dyspnea. The presence of the r.C50>A mutation in *TERC* in the germline genotype of III.2 was confirmed by sequencing a DNA sample isolated from a fibroblast biopsy taken at age 15 (Supplementary Table 1). At age 20, III.2 experienced DC-associated enteropathy with malabsorption. Her medical history also included multiple endocrinological insufficiencies, including hypogonadotropic hypogonadism with pubertas tarda, hypothyroidism, and osteoporosis. At age 21, she was notably small and dystrophic, at a height of 140 cm and a body weight of 30 kg. She died from a respiratory infection at age 21 following the rapid progression of pulmonary fibrosis.

The presence of the C50>A mutation in dizygotic twins indicated germline inheritance. Indeed, the mutation was also present in the mother (II.2) and her sister (II.3), strongly suggesting that the mutation had been present for at least one additional generation in this pedigree. It is presently unknown whether the previously identified father-son pair^19^ harboring the same mutation is also related to this family, which would imply a persistence of the mutation for at least five generations. While II.3 had a history of bronchial asthma that showed no evidence of restrictive lung disease, II.2 exhibited the first signs of DC at age 19, presenting with reticular hyperpigmentation on the neck. Although she never smoked, she was diagnosed with tongue squamous cell carcinoma at age 25. At age 40, she developed dyspnea and was subsequently diagnosed with idiopathic pulmonary fibrosis and DC. She was listed for lung transplantation at age 44 and underwent transplantation six years later. Her post-transplant course was complicated by CMV reactivation with pneumonitis, leukopenia, and anemia, findings attributed to a combination of immunosuppression, antiviral therapy, and underlying DC. One year after transplantation, she died of hemorrhagic shock following spontaneous retroperitoneal bleeding. In contrast, at age 59, II.3 exhibited blood counts within normal limits and no abnormalities in skin or nail morphology. At age 61, she was diagnosed with idiopathic pulmonary fibrosis.

### Clinical phenotypes are associated with short telomeres

Consistent with previous findings^19^, all individuals harboring the C50>A mutation in this study exhibited telomere lengths below the 10^th^ percentile in both lymphocytes (Fig. 1c) and granulocytes (Supplementary Fig. 1), with the younger generation of carriers, III.2 and III.3, exhibiting lymphocyte telomere length below the first percentile. Homozygous wild-type hTR offspring (III.1 and III.4) of mutant telomerase individuals present with normal blood counts and pulmonary function and no visible abnormalities of the skin or nails. However, while III.4 exhibited normal telomere lengths, III.1 showed markedly short telomeres below the first percentile.

Recent advances in telomere enrichment^22–24^ and computational analysis tools^25^ permit previously inaccessible telomere analysis using third-generation long-read sequencing on the Oxford Nanopore Technologies platform. Telomeres from II.3, II.4, and III.4 were sequenced using R10.4.1 MinION flow cells and basecalled with the Dorado v0.7.0 super-high accuracy basecalling model (v5.0.0). Telomeric reads were then analyzed using TARPON, which identifies reads containing full-length telomeric sequences based on the presence of both a terminal capture probe and a contiguous stretch of subtelomeric sequence. This approach yielded 6266 (II.3), 5464 (II.4), and 3007 (III.4) full-length telomeric sequences. As expected, III.4 had longer telomeres than both of her parents, likely reflecting her younger age and the homozygosity for wild-type *TERC* (Fig. 1d). Consistent with the presence of mutant telomerase, II.3 had shorter telomeres than her wild-type partner II.4. While the median telomere lengths measured by Nanopore sequencing were consistently shorter than those obtained by Flow-FISH (Fig. 1d, dotted lines), the two methods showed a strong correlation across the three samples (R² = 0.99, *p* = 0.01; Supplementary Fig. 2). A small fraction of reads in each sample contained very short telomeric tracts ( < 200 bp). It remains unclear whether these represent genuinely short telomeres or arose from fragmentation during DNA extraction, storage, or handling prior to ligation of the capture probe.

### RTEL1 nonsense mutation as a genetic modifier

In the absence of a mutant *TERC* allele in III.1, it was unclear why she exhibited shortened telomeres, while III.4 did not. A search of the III.1 WGS data for possible genetic modifiers revealed the presence of a Q1178x nonsense mutation in *RTEL1* (NM_001283009.2), a gene previously implicated in telomere biology disorders^26,27^. Even though this specific allele has not been reported in the literature, at least 71 nonsense mutations, predominantly distributed across the C-terminal half of RTEL1, are listed as pathogenic or likely pathogenic in ClinVar with at least one star, including several nonsense mutations downstream of position 1178 (Fig. 1e). For a variant to achieve the one-star status on ClinVar, the variant must be classified in a way that is consistent or more thorough than current practice guidelines and evidence or rationale for its variant scoring must be provided.

Further analysis of WGS data also revealed the presence of the RTEL1 nonsense mutation in individuals III.2 and III.3 (Fig. 1a and Supplementary Table 2). Although paternal DNA was not directly available for analysis, the Q1178x variant was detected in the blood of individual III.2, who had received a stem-cell transplant from II.1. In addition, the detection of the mutation in all three siblings, and its absence in II.2, II.3, and III.4, strongly supported paternal inheritance from II.1. Consistent with the mutant transcript being targeted by nonsense-mediated decay, RNA sequencing data revealed an allelic expression bias favoring the wild-type transcript in both III.3 (68% WT, 32% MT) and III.1 (76% WT, 23% MT). Consequently, total *RTEL1* mRNA levels were reduced relative to control samples (Fig. 1f). Given the established role of RTEL1 in telomere maintenance, the presence of this mutation could explain the pronounced difference in telomere length between III.1 (below 1^st^ percentile) and III.4 (above 50^th^ percentile). Notably, III.2 carried the RTEL1 germline mutation and received a paternal stem cells transplant carrying the mutation. Her extremely short telomeres (below the 1st percentile at age 20) were likely attributable to the mutation and further telomere shortening during engraftment^28^. Nevertheless, the paternal stem cell transplant corrected the hematopoietic deficiency, and no further hematologic abnormalities were documented during her lifetime.

### Reconstitution of mutant hTR

**Fig. 2:**
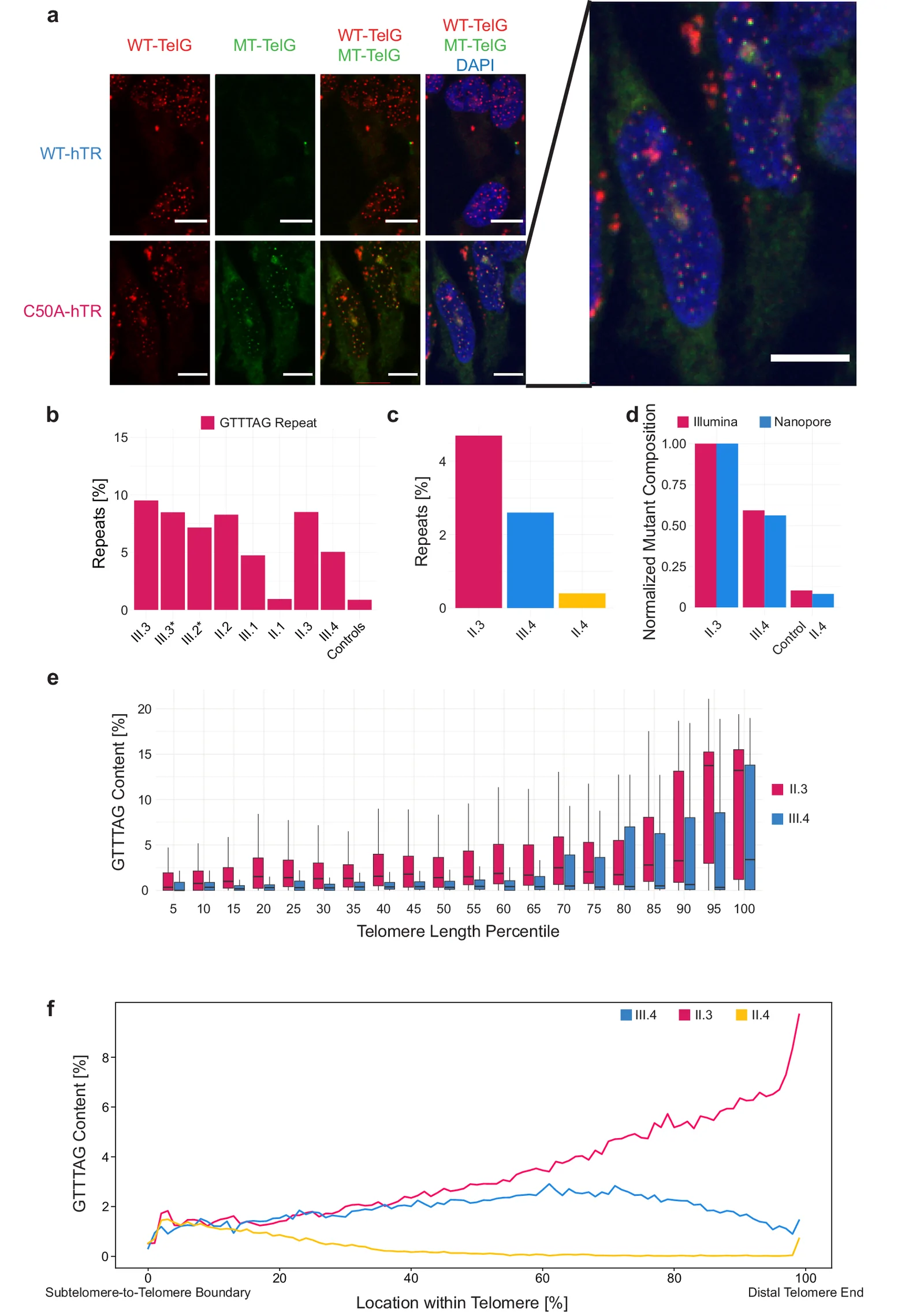
C50>A mutant telomerase forms an active complex in vitro and in vivo. **a** Transfected HeLa cell nuclei during interphase stained with DAPI (blue) and telomeric probes recognizing either wild-type sequence (TTAGGG)_3_ (red) or mutated sequence (GGTTTA)_3_ (green). Scale bars correspond to 10 μm. **b** The frequency of mutant telomeric repeats normalized to the number of base pairs in fully telomeric sequences per sample across all individuals sequenced in this study by Illumina techniques and (**c**) Nanopore sequencing. **d** Fraction of mutant telomeric repeats found within different sequencing techniques normalized to II.3. **e** Telomere length correlation with mutant repeat composition within telomeric sequences of patients II.3 and III.4 binned into five percentile groups by telomere length. The median is indicated by the solid horizontal black line. The hinges represent the 25th and 75th percentiles (the first and third quartiles), and the whiskers extend to the smallest and largest values within 1.5 × the interquartile range (IQR) of the lower and upper hinges, respectively. **f** Average mutant repeat composition in 1% increments of telomeric sequence from the subtelomere-to-telomere boundary to the distal telomere end in individuals II.3, II.4 and III.4.

While RTEL1 exhibited a decrease in the amount of total RNA and an allelic imbalance, wild-type and mutant hTR were present in equal proportions (Supplementary Table 1). To determine if hTR harboring the C50>A mutation can form a functionally active telomerase, HeLa cells were co-transfected with expression plasmids encoding hTERT and either wild-type or r.*C50*>*A TERC*. Reverse transcription of the mutant template by telomerase was expected to generate GTTTAG repeats. Telo-FISH was performed using probes specific for wild-type and mutant telomeric repeats. A probe for the mutant C-strand sequence (TAAACC)_n_ marked a distinct punctate nuclear pattern that colocalized with the signal for wild-type telomeres only in cells transfected with r.*C50*>*A TERC* (Fig. 2a). This co-localization is consistent with previous findings in hTERT-RPE cells^19^ and strongly indicates that the exogenously introduced C50>A RNA formed catalytically active telomerase complexes that extended native telomeres by at least three consecutive mutant repeats, followed by successful lagging strand synthesis, to permit hybridization of the (GGTTTA)₃ peptide nucleic acid probe to telomeres. The C50>A variant thus neither triggered immediate nucleolytic degradation of the newly synthesized G-strand, nor did it impair the synthesis of the C-strand in cultured cells.

To test whether the presence of mutant telomeric repeats was a result of acute over-expression of telomerase in cancer cells or was also observed under physiological conditions, patient whole genome sequencing (WGS) data was analyzed. The identification of variant repeats in telomeric reads isolated from WGS data demonstrated that the mutant telomerase RNA subunit was indeed incorporated into functional and catalytically active telomerase complexes in vivo (Fig. 2b). Although telomeric reads were predominantly composed of wild-type repeats (87%), the GTTTAG repeat accounted for 9.5% of telomeric sequences in III.3, well above the background level of variant repeats attributable to biological and technical noise. In two samples from unrelated donors harboring only wild-type *TERC*, the GTTTAG sequence represented approximately 0.8% of telomeric repeats, while wild-type GGTTAG accounted for 97%, with the remaining 2.2% representing other single-nucleotide variations or small insertions within the telomeric array (Supplementary Fig. 3). Notably, the GTTTAG sequence was almost 20-fold more abundant in telomeric reads from individual III.3 than the second most common one-nucleotide deviation from the wild-type repeat sequence (Supplementary Fig. 4a). Even though GTTTAG was also the most frequent non-canonical repeat in control samples harboring only wild-type *TERC*, it occurred only 2-fold more often than the next most prevalent variant sequence GGTTTG (Supplementary Fig. 4b).

Analysis of telomeric reads from the WGS data of II.2 and II.3 revealed a mutant repeat (GTTTAG) content of 8.3% and 8.5%, respectively (Fig. 2b), and no noticeable increase in mutant telomeric repeat content was detected with increasing generations. This is in direct contrast to previous findings where a marked increase in mutant telomeric repeat composition from the father (2.7%) to the son (9.2%) was reported^19^. This may suggest that the mutation is generationally younger within their family. Alternatively, it may represent differences in telomere dynamics between buccal epithelial cells and leukocytes, or differences in telomere inheritance between genders, as the mutation was paternally inherited in Hinchie et al.^19^ and maternally in this study.

The mutant telomeric repeat frequency in III.2 was first analyzed in the blood, as we were not aware of the stem cell transplant she had received from her father. Mutant telomeric repeat content was at background level (0.94%), and we now denote this sample as originating from II.1, as the hemopoietic system was reconstituted from paternal HSCs (Fig. 2b). Subsequent analysis of sequencing data derived from III.2 fibroblasts revealed that GTTTAG repeats represented 7% of telomeric sequence, 23.5-fold more frequent than the second most abundant variant repeat (*GGTTTG*). These statistics were slightly lower than those observed in blood samples of III.3, who inherited the same *TERC* and *RTEL1* mutations, and we wondered whether this reflected differences between tissues rather than individuals. Sequencing of fibroblasts from III.3 showed that telomeric repeat dynamics can indeed vary between tissue types. In fibroblasts from III.3, the mutant repeat frequency dropped from 9.5% to 8.5%.

### Inherited mutant repeats in wild-type individuals

Little is known about the dynamics of telomeric sequence turnover in the hemopoietic lineage across embryogenesis, childhood, and into adult life. To assess whether III.1 and III.4 harbored mutant telomeric sequences in peripheral blood leukocytes despite wild-type *TERC* homozygosity, we performed WGS on DNA isolated from blood. Based on the sequencing data for the respective mothers, we inferred that maternally inherited chromosomes carried ~ 8% mutant telomeric repeats at the time of conception, while telomeres on paternal chromosomes were composed of only wild-type repeats. This would place the starting frequency of mutant repeats in the fertilized oocyte at approximately 4%, assuming similar length for maternal and paternal telomeres. This estimate is conservative, particularly for individual III.4, whose mother’s telomere length was 4.8 kb at age 58, while her father’s (II.4) telomere length was 6.5 kb at age 62 (Fig. 1c). In the absence of the *r*.*C50*>*A TERC* allele in III.4, any telomere elongation after zygotic genome activation would have been mediated solely by wild-type telomerase, and we thus expected the percentage of mutant repeats to be substantially lower than 4%. It was therefore surprising that, after more than 25 years, the fraction of mutant repeats remains at ~4% (Fig. 2b). These findings suggest that little to no replacement of mutant repeats occurred on the maternal chromosomes during this entire period. A similar pattern was observed in individual III.1, although no data was available for paternal telomere length in this case.

To assess telomere turnover, we analyzed the positional distribution of mutant repeats in long-read telomere data. Telomeric sequences contained 4.7%, 2.6%, and 0.4% GTTTAG mutant repeats in II.3, III.4, and II.4 respectively (Fig. 2c). The relative difference in mutant repeat content between individuals is consistent across sequencing platforms: the mutant composition decreases between II.3 and III.4 by a factor of 0.56 for Nanopore and 0.59 for Illumina data (Fig. 2d). This trend is also apparent when comparing the telomeres of III.4 to those of her father II.4, or in the case of the Illumina data, to an age-matched control. The apparent difference in absolute mutant repeat content is explained by the inclusion of the variable region at the subtelomere to telomere transition in the ONT data, whereas Illumina datasets were filtered for bona fide telomeric reads.

The availability of long-read data permitted us to examine the relationship of telomere length and mutant telomeric repeat content. Surprisingly, when telomeres were binned by length into 5% increments, and the mutant repeat content was calculated for each bin, a clear positive correlation was apparent, with longer telomeres containing more mutant repeats in both II.3 and III.4 (Fig. 2e). This finding was unexpected in light of the severely reduced processivity of the C50>A mutant described previously^19^.

Long-read sequencing also provided positional information along individual telomeres that was not accessible from Illumina data. Telomeric sequences were divided into 1% intervals and the average GTTTAG repeat frequency per interval was calculated across all reads and plotted from the subtelomere-to-telomere boundary to the distal end of the telomeric read (Fig. 2f). In individual II.4, who carries only wild-type telomerase, the GTTTAG signal peaks at the subtelomere–telomere boundary, reflecting the presence of mutant telomeric repeats within the telomere variant repeat (TVR) region, and declines to 0% towards the distal end of the telomere. In contrast, individual II.3, who is heterozygous for the C50>A mutation, exhibited a progressive increase in mutant repeat frequency from the TVR to the distal end, consistent with ongoing incorporation of mutant repeats by the variant telomerase. For individual III.4, who inherited only wild-type TERC alleles, mutant repeat frequency also increased beyond the TVR but gradually declined toward the distal end of the telomere. These opposing patterns in mother and daughter are consistent with C50>A telomerase adding mutant repeats at the ends of telomeres in II.3, whereas newly synthesized distal repats are exclusively wild-type in III.4. However, the distal telomere ends in III.4 do not exhibit GTTTAG repeat levels as low as those observed in II.4. Reads terminating in mutant repeats likely represent chromosome ends for which wild-type telomeric repeats added early in life have eroded through successive rounds of replication, until they now terminate in a tract of mutant repeats inherited from the mother.

Notably, the most distal 1% of telomeric reads in II.4 and III.4 contain a higher fraction of mutant telomeric repeats than expected. These mutant repeats, particularly evident in II.4, are predominantly confined to the most distal telomeric repeat sequenced, which reveals an artifact of the telomere enrichment methodology. During enrichment, capture probes are intended to anneal to the G-strand overhang in a manner that favors ligation to the termini of the G-strand or C-strand, respectively. However, the sequencing data revealed that capture probes also anneal to the G-strand overhang while leaving a gap, that is then filled in by the end-preparation and nick repair steps during library preparation (Supplementary Fig. 5a). Such fill-in synthesis resulted in the appearance of a terminal mutant repeat even in wild-type control samples. This partial annealing, followed by fill-in synthesis, became apparent here due to the use of capture probes containing mixtures of wild-type and mutant repeats. We note that this observation renders the methodology unsuitable for determining the terminal sequence of telomeres.

### C50>A telomerase exhibits decreased processivity

**Fig. 3:**
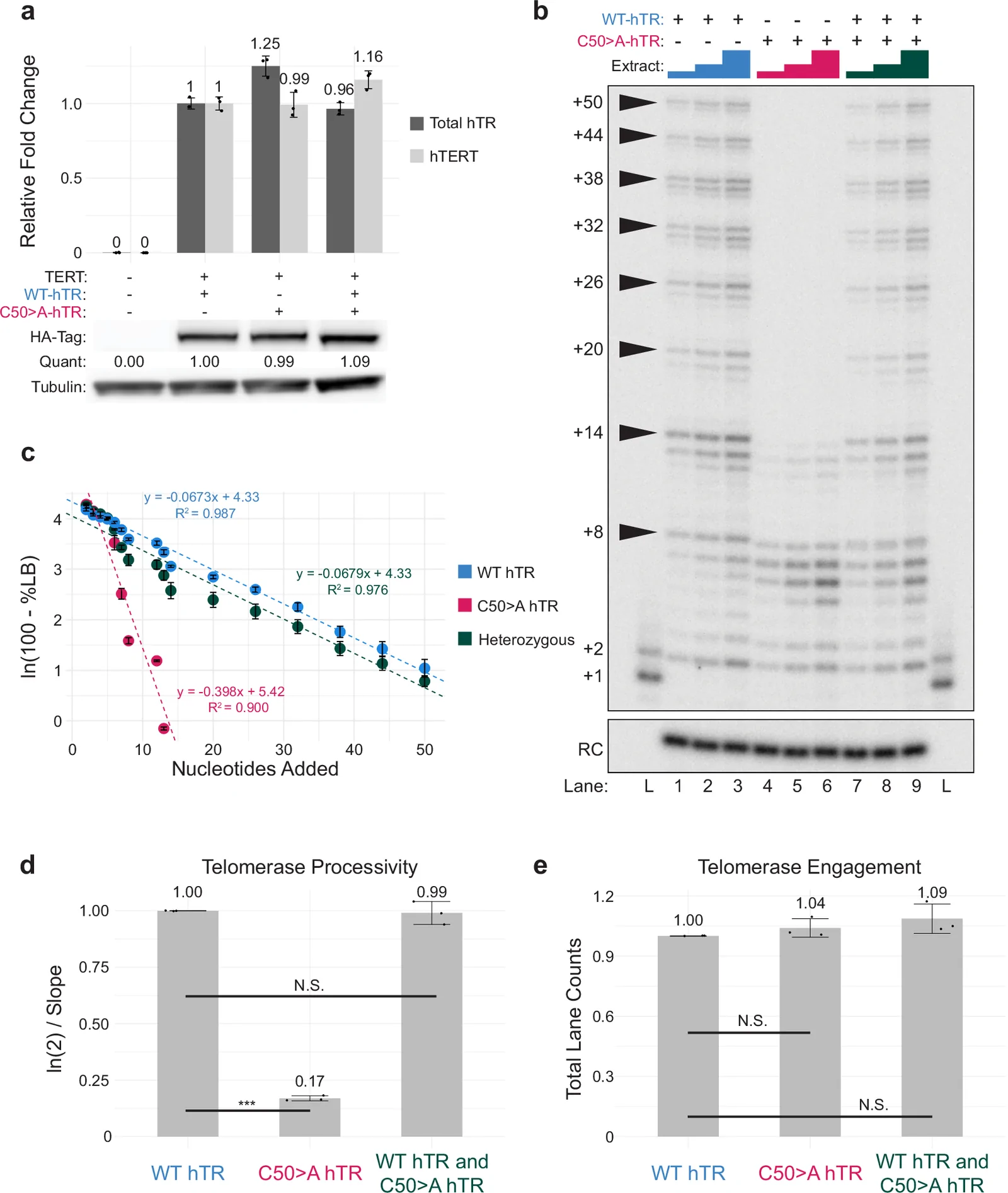
*C50*>*A* hTR mutant telomerase is severely unprocessive without affecting its initial engagement. **a** qPCR analysis (upper) and western blot analysis (lower) for the relative levels of overexpression for hTR, *hTERT* mRNA, and hTERT isolated from cellular extracts used for the DTAA in panel b. Values are normalized to the samples overexpressing *hTERT* and WT-hTR; bar height represents the mean of the technical replicates. Error bars denote ± 1 standard deviation (SD) centered on the mean. The relative quantification of the 3xHA-hTERT western blot band is normalized to the signal of the respective tubulin band. Primers for hTERT anneal in exon 1 and exon 2, respectively. Endogenous *hTERT* mRNA is below the limit of detection under the conditions used here. **b** DTAA products from either 3 µg, 6 µg, or 12 µg of VA13 cell extract expressing: wild-type hTR, C50>A hTR, or a 1:1 combination of both constructs with wild-type telomeric primer (AGGGTT)_3_; L = Ladder; RC = Recovery Control. **c** Telomerase activity quantification of the gel in panel b as described in Materials and Methods. Different concentrations of the same extract are treated as independent technical replicates. Error bars equal ± 1 standard deviation (SD) among technical replicates. **d** Processivity calculations resulting from the slopes of panel (**c**) as described in Materials and methods and normalized to wild-type hTR. Bar height represents the mean of the technical replicates. Error bars denote ± 1 standard deviation (SD) centered on the mean (*** = *p* < 0.001 calculated with T. student statistical analysis). **e** Telomerase engagement quantification of DTAAs (three biological replicates: panel **b**). The TLCs of each lane were calculated as described in Materials and Methods and normalized over wild-type hTR samples. Bar height represents the mean of the technical replicates. Error bars denote ± 1 standard deviation (SD) centered on the mean.

To assess whether the mutant telomerase was indeed less processive than its wild-type counterpart, we performed direct telomerase activity assays (DTAAs). WI-38 VA13 cells were used due to their known absence of detectable endogenous *TERC* expression^29,30^, and were transfected with *hTERT* and either wild-type or r.*C50*>*A TERC* or a 1:1 combination of both constructs to mimic the heterozygous state of the patient. Quantitative PCR and Western blotting confirmed similar expression levels of *hTERT* mRNA and *TERC* across all samples (Fig. 3a). Telomerase activity assays produced the characteristic 6-nucleotide banding pattern for the wild-type enzyme (Fig. 3b, lanes 1–3). In sharp contrast and similar to findings by the Alder group^19^, extracts containing only C50>A telomerase generated a distinct pausing pattern and only short extension products indicative of low processivity (Fig. 3b, lanes 4–6). A 1:1 mixture of both wild-type and *r.C50*>*A TERC* produced an overlay of the two patterns (lanes 7–9).

To further characterize differences in repeat addition processivity, total lane counts (TLCs) were calculated by summing the intensity of each visible extension product, normalized to the number of incorporated radiolabeled guanosines and to the loading control. The percentage left behind (%LB) for each product *n* was determined by summing the intensities of all products shorter than *n*, adding the intensity of band *n*, dividing by the TLC, and multiplying by 100. As a measure of processivity, ln(100−%LB) was then plotted against the number of nucleotides added by telomerase (Fig. 3c) and telomerase processivity was calculated as ln(2) divided by the slope of the linear regression (Fig. 3d). This analysis confirmed that C50>A telomerase exhibited a 5-fold reduction in processivity compared to the wild-type enzyme, consistent with previous findings^19^. Notably, the mixture of both wild-type and C50>A hTR displayed processivity equivalent to that of wild-type hTR, indicating that the presence of mutant RNA did not affect the processivity of the wild-type enzyme under single turnover conditions (Fig. 3d). In contrast to the low processivity of C50>A telomerase, its rate of initial engagement with the telomeric primer was indistinguishable from wild-type telomerase (Fig. 3e). This observation is consistent with the C50>A mutation residing outside the alignment region where the stabilizing base-pairing interactions between telomerase RNA and the telomeric primer occur.

**Fig. 4:**
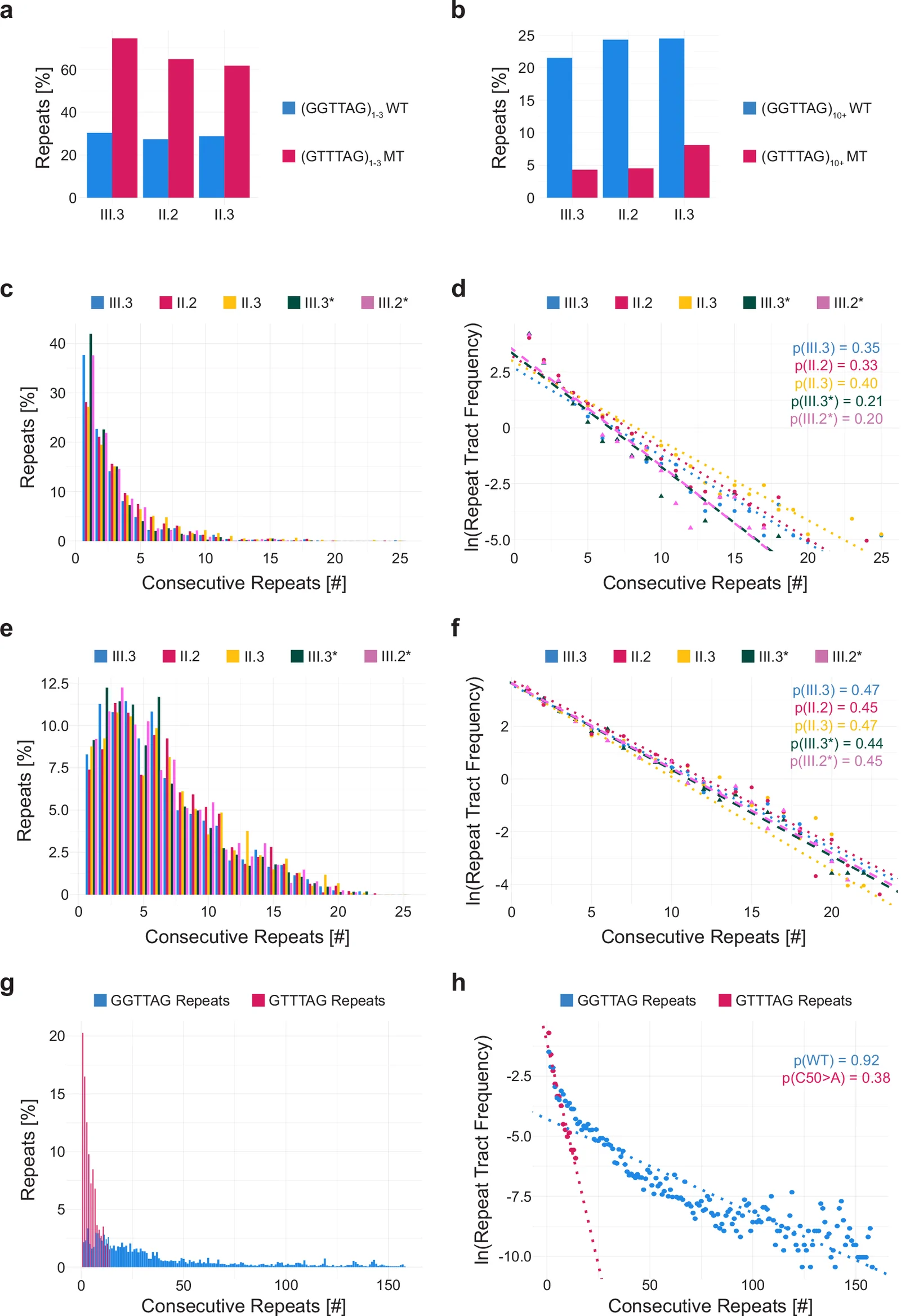
Decreased *C50*>*A* processivity observed in vivo. **a** Percentage of telomeric repeats found in repeat tracts of less than 4 consecutive repeats and (**b**) the percentage of telomeric repeats found in repeat tracts of 10 or greater consecutive repeats. **c** The percentage of mutant telomeric repeats found in a given consecutive tract length from Illumina WGS of blood or *fibroblasts. **d** The natural log frequency of mutant repeat tracts plotted against the length of repeat tracts to calculate mutant repeat processive addition probabilities. **e** The percentage of wild-type telomeric repeats found in a given consecutive tract length from Illumina WGS of blood or *fibroblasts. **f** The natural log frequency of wild-type repeat tracts plotted against the length of repeat tracts to calculate wild-type repeat processive addition probabilities. **g** Percentage of mutant and wild-type telomeric repeats by tract length in the terminal 1 kb of telomeres sequenced from patient II.3 and the (**h**) natural log frequency of repeat tract counts used to calculate processivity.

To assess telomerase processivity in vivo, we further analyzed telomeric sequences derived from the patients’ Illumina whole genome sequencing data. Consistent with a decreased processivity of mutant telomerase in vitro, greater than 60% of mutant telomeric repeats were found in tracts of fewer than four repeats across all blood samples (Fig. 4a). In contrast, fewer than 35% of all wild-type repeats are found in such short repeat tracts (Fig. 4a), and greater than 20% of wild-type repeats are found in repeat tracks of ten or more repeats in all blood samples (Fig. 4b).

A linear regression of repeat number versus their log-transformed frequency was used to calculate the probability of processive repeat addition as detailed in the Methods section. Processivity, here defined as the probability that a specific repeat was added by the same enzyme as the previous repeat, was 0.35, 0.33, and 0.40 from the blood of individuals III.3, II.2, and II.3, respectively. The higher processivity in vivo is likely due to the presence of the telomerase processivity promoting factors TPP1 and POT1^31^. However, apparent processivity of mutant telomerase was lower in fibroblast derived samples for both III.3 (0.21) and III.2 (0.20) (Fig. 4c, d), an observation that hints at tissue-specific differences in telomerase activity or telomere dynamics. The apparent processivity for wild-type telomerase was only modestly higher than the mutant (Fig. 4e, f), but this analysis underestimated wild-type enzyme processivity due to the 150-nucleotide Illumina read-length limitation, which imposed an artificial upper boundary on the available repeat tract length data. The magnitude of this effect is clearly evident as 49% of all telomeric reads from the blood of III.3 consisted exclusively of wild-type repeats and were thus removed from the analysis, as repeat tract length can only be calculated if wild-type repeats are flanked on both sides by mutant repeats. Moreover, for reads containing both wild-type and mutant sequences, 77% of read boundaries occurred within wild-type tracts. In contrast, no reads were composed solely of mutant repeats.

In aggregate, these observations make short-read sequencing data of limited value for processivity calculations for wild-type telomerase. Long-read sequencing data addresses this shortcoming by permitting identification of the boundaries of stretches of consecutive wild-type and mutant repeats. Continuous stretches of wild-type repeats were identified by isolating sequences flanked on both sides by at least one GTTTAG repeat. Stretches of mutant repeats were identified in the corresponding fashion. To reduce the influence of “older” internal telomeric sequences, including the telomere variant repeat (TVR) regions that have accumulated point mutations during normal DNA replication or DNA repair, only the terminal 1 kb of each telomere was included in this analysis. To further eliminate TVR-regions from very short telomeres, the data was further filtered to only include sequences that were comprised solely of GGTTAG and GTTTAG repeats. As expected, mutant repeat tracts were considerably shorter than wild-type tracts. Specifically, 49.3% of mutant repeats were identified in groups of one to three repeats, compared to only 7.8% of wild-type repeats (Fig. 4g). Processivity was calculated by plotting the natural log of the number of repeat tracts against their length, using the same method as previously applied to Illumina WGS data. At 0.38, the calculated apparent processivity of mutant telomerase was the same as calculated from Illumina data. In contrast, the apparent processivity for wild-type telomerase was substantially higher when based on the long-read sequencing data (0.92 compared to 0.47 in the Illumina data). In addition, long-read sequencing suggests that wild-type telomerase may exhibit bi- or tri-phasic processivity behavior with a steeper slope at shorter repeat stretches that lessens as consecutive repeat number increases (Fig. 4h). This observation highlights the value of long-read sequencing data in characterizing telomerase activity in vivo and warrants further investigation.

### Analysis of enzyme processivity and sequence heterogeneity

**Fig. 5:**
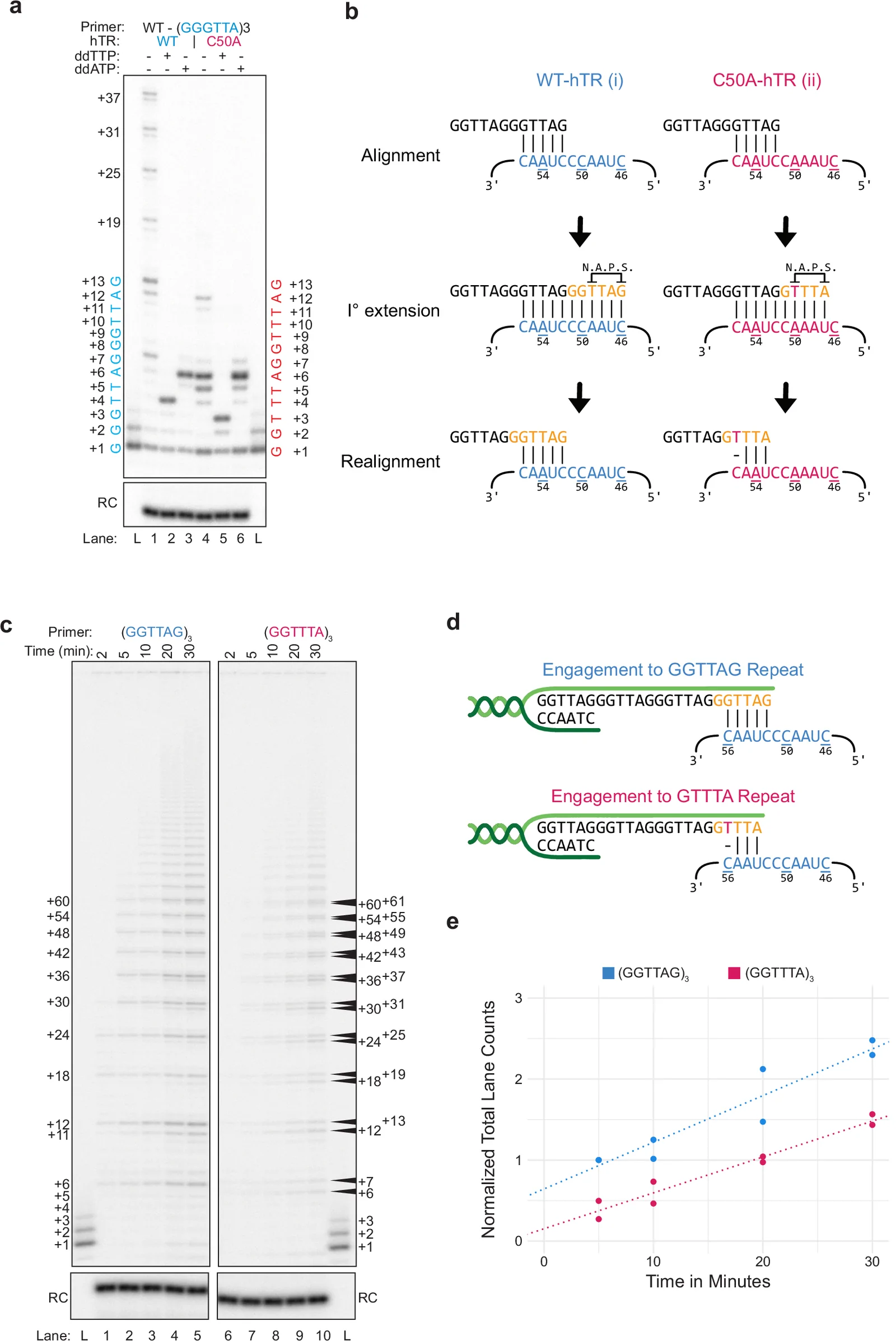
Aberrant telomere elongation by C50>A hTR mutant telomerase and mutated telomeric substrates. **a** Amplification products of a DTAA using 6 µg of VA13 cell extract, expressing either wild-type or C50>A hTR and extending a wild-type template. dATP or dTTP were replaced with the respective di-deoxynucleotide analog, where indicated (L = Ladder, RC = Recovery Control). **b** Schematics showing the elongation of wild-type template sequences by wild-type and C50>A mutant telomerase as predicted by the DTAAs shown in panels (**a**). **c** Products of a time course DTAA using 6 µg of VA13 cell extract expressing wild-type hTR with wild-type and mutant telomeric primer as indicated (L = Ladder, RC = Recovery Control). **d** Alignment of wild-type telomerase to wild-type GGTTAG or mutant GTTTA repeats as seen in panel (**c**). **e** Telomerase activity quantification of two biological replicates of DTAAs using 6 µg of extract, as shown in panel c. The TLCs of each lane were calculated as described in Materials and Methods and normalized to wild-type hTR samples at 5 min.

To investigate the mechanistic basis for the reduced processivity of C50>A telomerase, dATP and dTTP were replaced with the corresponding chain-terminating dideoxynucleotide analogs in separate in vitro activity assays (Fig. 5a). When elongating a primer ending in GGGTTA, both wild-type and mutant telomerase initially added a single guanosine ( +1 band, Fig. 5a, lanes 1–6). In the absence of chain terminators, wild-type telomerase then translocated and extended the product by an additional six nucleotides ( +7 band, lane 1). Substitution of dTTP with ddTTP resulted in termination after the addition of four nucleotides (G–GGT; lane 2), and replacement of dATP with ddATP yielded the expected six-nucleotide extension product (G–GGTTA; lane 3). Consistent with the mutant enzyme using the same alignment register, ddTTP resulted in a three-nucleotide extension (G–GT; lane 5), and ddATP permitted synthesis of a six-nucleotide product (G–GTTTA; lane 6).

Most interestingly, in the absence of chain-terminating nucleotides, C50>A telomerase added only six nucleotides (G-GTTTA; lane 4), rather than the seven observed with the wild-type enzyme (lane 1). This observation is consistent with a previously reported nucleotide addition pause site three nucleotides downstream of the first adenosine in the RNA template^32^. The altered template sequence in C50>A shifts the nucleotide addition pause signal (NAPS) by one nucleotide, consistent with termination of nucleotide addition following reverse transcription of position 47 rather than position 46 (Fig. 5b, compare panels (i) and (ii)). This generates an extension product ending in the sequence -GTTTA-3’ for C50>A versus GGTTAG-3’ for wild-type telomerase and promotes the dissociation of telomerase rather than its processive extension.

We reasoned that the disease phenotypes may be explained solely by reduced enzyme processivity, but the variant telomere sequences may also contribute by weakening the interaction between the alignment region of wild-type telomerase and a variant terminal repeat in the telomere. To assess whether terminal variant repeats impaired engagement by wild-type telomerase, in vitro telomerase assays were conducted using wild-type (GGTTAG)₃ and variant (GGTTTA)₃ primers. The variant primer consistently yielded fewer extension products than the wild-type primer (Fig. 5c, compare lanes 1–5 with lanes 6–10), consistent with a shorter annealing region as illustrated in Fig. 5d. Quantification of extension products showed that engagement of wild-type telomerase with the variant primer was reduced by two- to three-fold relative to the wild-type sequence (Fig. 5e). However, once the initial round of binding and extension had occurred, the wild-type primer yielded the expected +6 nucleotide ladder (Fig. 5c, lanes 1–5), whereas the variant primer produced both +6 and +7 nucleotide products resulting from alternative alignments (see below). Although both primers were extended with the same processivity, the variant primer reduced initial engagement for wild-type telomerase.

### Sequence specific junctions of telomeric repeats

**Fig. 6:**
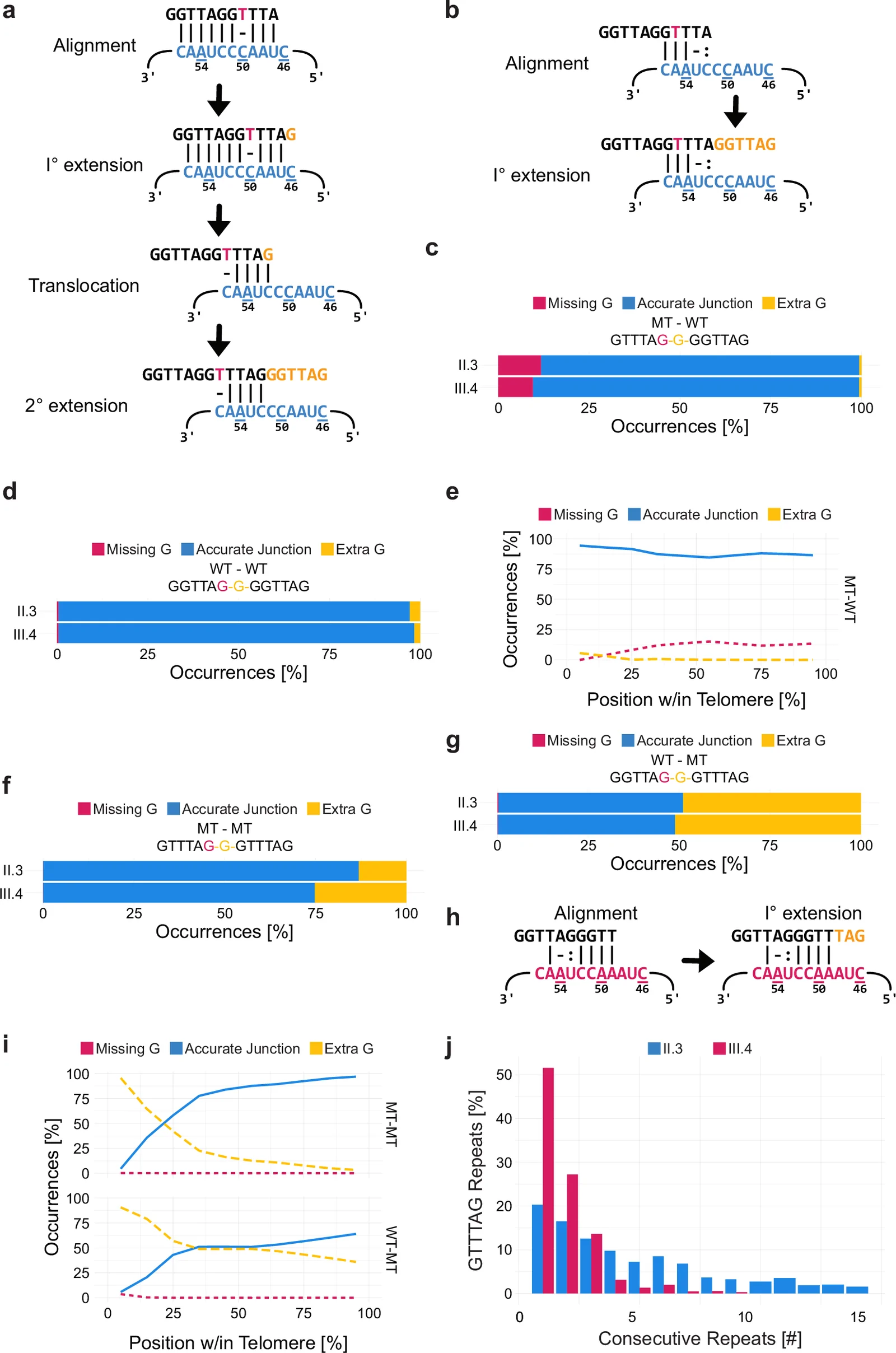
Variation in sequence junctions between consecutive repeats. **a** Schematic depicting wild-type telomerase extension of a mutant telomeric repeat and (**b**) an alternative alignment that would result in the absence of an internal guanosine residue. **c** The frequency of MT-WT repeats that are lacking an internal guanosine residue or contain an additional guanosine residue in Nanopore Telomere sequencing of II.3 and III.4. **d** The frequency of guanosine insertions and deletions between two consecutive wild-type telomeric repeats. **e** The location of accurate junctions or junctions containing insertions or deletions of MT-WT repeat boundaries in nanopore telomere sequences of II.3. Position is determined by dividing every telomere into 10% increments of sequence from the subtelomere-to-telomere boundary to the end of the telomere. **f** The same as c for MT-MT boundaries, and (**g**) WT-MT boundaries and (**h**) a hypothetical alignment that could explain the insertions observed between WT and MT repeats. **i** The location of MT-MT (top) and WT-MT (bottom) consecutive repeats in II.3 Nanopore telomere sequences divided as described in (**d**). **j** The percentage of mutant telomeric repeats found in a given repeat tract length comparing II.3 to her daughter III.4.

In vitro telomerase assays indicated that C50>A telomerase preferentially dissociates following the addition of the sequence GTTTA (Fig. 5a). This new terminus can then engage with wild-type telomerase in two registers, neither involving perfect base pairing (Fig. 6a, b). Analysis of the Illumina and Nanopore telomeric sequence data suggested that the 10-nucleotide alignment shown in (a) constitutes the majority of events in vivo. Reverse transcription of C46 is then followed by translocation (Fig. 6a). However, ~12% of mutant repeats followed by a wild-type repeat were missing one G at the start of the wild-type repeat (Fig. 6c), supporting the alternative alignment (Fig. 6b). Here, reverse transcription of template positions 51 to 46 generates two guanosines followed by -TTAG prior to the next translocation event. This sequence TTA-GGTTAG is specific to the transition from mutant to wild-type repeat and is absent when wild-type telomerase extends wild-type telomeric repeats (Fig. 6d). In addition, MT-WT junctions found within individual III.4, who inherited mutant telomeric repeats but only wild-type TERC, exhibit a similar fraction of the TTA-GGTTA junctions (Fig. 6c). Furthermore, long read sequencing data showed that the incidence of these junctions is constant along the length of telomeres outside of the proximal telomere variant regions (Fig. 6e).

When examining MT-MT (Fig. 6f) or WT-MT (Fig. 6g) junctions, an additional guanosine residue was detected 13% (GTTTAG-**G**-GTTTAG) and 49% (GGTTAG-**G**-GTTTA) of the time in telomeres from II.3. While we cannot exclude that the additional guanosine residue between WT and MT repeats may result from an alternative alignment of hTR (Fig. 6h), the frequency of MT-MT insertion events decreases strongly towards the distal end of the telomere (Fig. 6i). This finding suggests that the additional G may not be attributable to telomerase action, but is instead the result of “slippage” during DNA replication. This hypothesis is further supported by the observation that G insertions are highest in the telomeres of III.4, an individual who only harbors wild-type telomerase. Mutant repeats present within her telomeres would have undergone many rounds of replication during embryogenesis. While the incidence of G insertions between wild-type and mutant repeats is similar between II.3 (48.9%) and III.4 (51.1%), G insertion events between two MT repeats nearly doubled between mother (II.3; 13.1%) and daughter (III.4; 25.2%), further suggesting that the pattern results from DNA replication, not telomerase action (Fig. 6f).

If the observed guanosine insertions originated from DNA replication slippage, the mutant repeat tract length should be shorter in III.4 compared to her mother (II.3). Indeed, long-read sequencing data shows a clear shift in the distribution of repeat tract length between mother and daughter towards shorter tract length (Fig. 6j). While 49% of mutant repeats occur as singlets or in groups of two or three repeats in II.3, over 90% of mutant repeats fall into this category in the telomeres of her daughter (III.4). In aggregate, these observations strongly suggest that repeat tract lengths, and thus apparent processivity of the mutant enzyme, is altered by conventional DNA replication causing repeat contraction over time. These observations suggest another, and perhaps unexpected, mechanism by which mutant telomere sequences contribute to accelerated telomere shortening even in individuals who have not inherited the C50>A mutation.

## Discussion

In this study, we present a comprehensive molecular and clinical analysis of the *r.C50*>*A* mutation within the template region of *TERC*. Our results show that this single-nucleotide variant disrupts telomerase function primarily through reduced processivity, supporting the mechanistic basis for the disease phenotype proposed for another case with the same mutation^19^. In addition, we show that terminal mutant repeats also impair subsequent engagement of wild-type telomerase. Whole genome sequencing and long-read telomere profiling revealed that the C50>A mutation is associated with ~8% mutant repeats in the telomeres of heterozygous carriers. This fraction is suprisingly similar across four individuals representing two generations. Strikingly, a mutant repeat content of 4% in two individuals who did not inherit the C50>A mutation suggests that telomeres are rarely subject to cycles of extensive shortening and subsequent re-elongation, as these would have replaced mutant with wild-type repeats. The telomere length equilibrium may thus be maintained in a narrow window across generations and over decades of cell divisions, at least in the hematopoietic lineages.

The identification of an RTEL1 nonsense mutation in a subset of family members provided a plausible genetic basis for the marked interindividual variation in telomere length and disease severity among family members. While this mutation alone was not associated with DC in individuals II.1 and III.1, its presence in combination with the C50>A variant appeared to exacerbate telomere shortening and dysfunction, as supported by the severe bone marrow failure and pulmonary fibrosis observed in individuals III.2 and III.3. These findings underscore the importance of considering secondary genetic modifiers or oligogenic contributions when evaluating patients with telomere biology disorders^33^.

### Evolutionary context and impact of template mutations on telomere function

The telomeric GGTTAG repeat has been evolutionarily conserved in vertebrates for over 400 million years^18^, reflecting its critical role in chromosome end protection and genome stability. This deep evolutionary conservation likely emerged as a compromise between telomere stability and the functional requirements of the telomerase reverse transcriptase^34^. Even minor deviations from this canonical sequence can have profound biological consequences, as telomeric sequence variation affects the binding affinities of several shelterin components, which in turn impacts both the regulation of telomerase access and chromosome end protection^1^. A comprehensive analysis of template mutations affecting the TTA part of the repeat documented diverse effects on extension rates and processivity in vitro^35^. In contrast, template mutations in *TERC* that disrupt GGG often compromised chromosome end capping and led to heterodicentric chromosome fusions^36,37^. Taking advantage of detrimental effects arising from the addition of some variant telomeric repeats, expression of telomerase template mutations in human cancer cells has been shown to reduce telomere length, impair growth, and increase apoptosis^38–40^. But not all deviations from the canonical repeat sequence impair telomere function. A screen of variant template sequences for the absence of cytotoxic effects identified the Tolerated Sequence 1 (TSQ1) template, which resulted in the addition of GTTGCG variant repeats, which were surprisingly well tolerated in a variety of cell lines^41^.

While the effects of introducing template mutations into cultured cells range from well tolerated to highly cytotoxic, only a small number of naturally occurring template mutations have been identified in humans thus far. To date, three pathogenic template mutations have been linked to DC or IPF in three families^42^. The A48>G substitution lies within the reverse-transcribed region and is predicted to alter the telomeric repeat sequence synthesized by telomerase to produce GGTCAG repeats. While initial functional analysis showed that A48>G severely compromised telomerase activity and exerted a dominant-negative effect on wild-type telomerase activity in vitro^43^, subsequent studies found that A48>G behaved as a loss-of-function allele in cell–based assays, with little or no evidence for dominant-negative interference^44,45^.

The Δ52–55 mutation removes four of the five nucleotides of the template alignment region (CUAA), a segment essential for the correct positioning of the telomeric 3’ overhang prior to extension. This deletion is therefore thought to impair telomerase activity by destabilizing primer alignment rather than altering repeat sequence, consistent with the severe activity defects observed in vitro^43^.

In both families, the parents of affected individuals were asymptomatic, and it remained unclear whether the mutations had arisen de novo in the affected individuals or represented cases of incomplete penetrance or transgenerational progression. Together, these cases underscore both the rarity of naturally occurring template mutations and the mechanistic diversity by which they can compromise telomerase function, ranging from altered repeat synthesis to profound defects in primer alignment, providing an important framework for interpreting the biochemical and clinical consequences of the template mutation described here.

The C50>A mutation described here and by Hinchie et al.^19^ results in DC and IPF. The previous study described a male who developed IPF at the age of 43 and subsequently received a lung transplant. His son also carried the mutation, and his telomeres harbored approximately 9% mutant repeats. At the age of 17, he was asymptomatic, consistent with the late onset of pulmonary fibrosis we report here for two C50>A germline carriers with a mutant repeat content of approximately 8%. It is presently unclear whether the mutation arose independently in the two families or whether they share a common ancestor. Hinchie et al. reported an imbalance in mutant and wild-type hTR detected through long non-coding RNA sequencing^19^. While spontaneous reversions of mutations within *hTERC* have been observed through mitotic recombination in families affected by DC^46^, this does not appear to be the case in the family described here, as high-coverage total RNA-sequencing evidence showed that the C50>A mutant and wild-type hTR were present in equal proportions.

### Molecular effects of a telomerase template mutation

The template mutation described here provided a unique opportunity to explore telomerase processivity in vivo. The long read sequencing data clearly demonstrated a genomic signature common to the telomeres of all carriers. Mutant repeat tracts were substantially shorter than wild-type repeat tracts, consistent with reduced processivity of the mutant telomerase in cells. While the overexpression of the TPP1-POT1 complex, factors that promote telomerase activity, slightly rescued the observed processivity decrease, it did not do so to the extent it improves wild-type telomerase processivity^19^. In addition, when a telomere was identified to end in a mutant telomeric repeat, GTTTA was the most commonly identified end^19^, consistent with in vitro DTAA findings reported here, and would therefore impair the ability of wild-type telomerase to extend telomeres.

While the template mutation results in decreased telomerase engagement and processivity, the mutant telomeric repeat has also been shown to reduce the binding affinity of POT1^19^. This reduced POT1 binding may increase telomerase accessibility at mutant-containing telomeres, contributing to the long, mutant-rich telomeres observed in this study. Despite the high sequence specificity of TRF1 and TRF2^47^ and the decreased binding affinity of POT1, the presence of the mutant telomeric repeat did not lead to an increased DNA damage response in patient-derived cells^19^. This suggests that the shelterin complex retains sufficient protective function at telomeres containing mutant repeats. The relatively low processivity of C50>A telomerase is likely to limit disruption of TRF1 and TRF2 binding, as the homodimers can bridge mutant tracts and interact with flanking wild-type telomeric repeats, as demonstrated in vitro for TRF1 binding two AGGGTT sites separated by non-telomeric DNA^47^. A detrimental effect of the mutant repeats on shelterin binding might be further mitigated by the guanosine insertions described here, as they generate additional higher-affinity binding sites for TRF1/2.

### Compounding effect of C50 > A TERC and RTEL1 mutations

The C50>A hTR template mutation was associated with the development of IPF in later adulthood in the patient described previously^19^ as well as individuals II.2 and II.3 described here. Although no medical records are available, reports from surviving relatives suggest that individual I.1 (mother of II.2 and II.3) also had late-onset disease, described as ‘strange nails’ and ‘bad blood counts’. Earlier onset and more severe clinical phenotypes, including bone marrow failure, were observed in generation III, but the interpretation of anticipation^48^ is confounded by the co-occurrence of the RTEL1 nonsense mutation in both individuals.

RTEL1 is crucial for the maintenance of genome stability, the regulation of telomere length, and the inhibition of homologous recombination at telomeres^21,27^. RTEL1 mutations have been associated with pulmonary fibrosis, dyskeratosis congenita, and Hoyeraal-Hreidarsson syndrome^49^. Disease-associated nonsense mutations most commonly occur alongside a missense mutation in the second allele^26,50^. However, a dominant effect has been documented in at least one family, where two siblings inherited a premature stop codon in RTEL1 from their mother, who had short telomeres. Both individuals developed Hoyeraal Hreidarsson syndrome^26^. In the cases described here, transcripts originating from the Q1178x allele were less abundant than wild-type transcripts (Supplementary Table 1), likely due to nonsense-mediated decay. Whether the clinical outcome is due to haploinsufficiency or whether the residual Q1178x mRNA results in the production of a protein with dominant negative properties is presently unclear. A comparison between III.1 and III.4 suggests that the RTEL1 mutation may impact telomere length. Neither individual inherited the *TERC* mutation, but both inherited mutant telomeric repeats from their respective mothers. Whereas telomeres of III.1 at age 30, a carrier of the paternal *RTEL1* mutation, are very short ( < 1^st^ percentile), telomere length for III.4 at age 27 was around the 50^th^ percentile.

Considering our data in aggregate, we conclude that the primary molecular cause of the short telomeres in individuals carrying the C50>A hTR mutation is insufficient telomere elongation caused by the combined effects of reduced processivity of the mutant enzyme and impaired elongation of telomeres terminating in the mutant sequence by both wild-type and mutant telomerase. When the C50>A hTR mutation was compounded with Q1178x in *RTEL1*, a severe clinical phenotype manifested early in life. These results underscore that comprehensive genetic evaluation, extending beyond single-gene diagnoses, is critical for understanding disease variability in telomere biology disorders and for informed assessment of affected families. Most notably, the inheritance of mutant repeats and their persistence into adulthood in an individual who harbored only wild-type telomerase argues that telomere maintenance occurs in a narrow window, and substantial shortening of telomeres followed by re-longation is a rare event.

## Methods

### Ethical approval and consent to participate

This study has been reviewed and approved by the Clinical Ethics Committee (CEC) of the Johannes Gutenberg-University Medical Center. Due to the fact that no clinical intervention or no direct patient-related procedures were performed, and our research is characterized as basic research, ethics approval by other authorities, such as the Ethics Commission of the Chamber of Physicians, is not applicable. All patients were included in the aplastic anemia bone marrow failure registry (AA-BMF, *EK332/20*, *EK225/14;* RWTH Aachen University). All procedures of the study involving human participants are conducted in accordance with the Declaration of Helsinki, applicable national laws, and institutional ethical guidelines.

All participants, or their legal guardians where appropriate, provided written informed consent prior to enrollment. Consent discussions emphasized the scope of the study, including the use of genomic and telomere sequencing technologies, the collection and long-term storage of donated biological samples, the potential risks and benefits, and the right to withdraw at any time without impact on medical care. Specific attention was given to the possibility that genomic sequencing could uncover secondary or incidental findings.

The CEC confirms that the study design appropriately safeguards participant autonomy, privacy, and confidentiality. Risks to participants are minimal and are outweighed by the potential benefits of advancing knowledge on the genetic and molecular basis of dyskeratosis congenita and related telomere biology disorders.

Consent for publication was obtained for the data relating to all individuals in this study. This study was conducted in accordance to the criteria set by the Declaration of Helsinki.

### Cell culture and transfections

WI-38 VA13 cells (ATCC-CCL-75.1) and HeLa cells (ATCC-CCL-2.2) were grown in complete medium [EMEM (Fisher Scientific, #11440335), 10% fetal bovine serum (LGC standards, #ATCC-30-2020) and 2 mM L-Glutamine (Fisher Scientific, #25030081)]. Fibroblasts derived from skin biopsies were grown in IMDM [(Fisher Scientific, #12589059), 15% fetal bovine serum (LGC standards, #ATCC-30-2020) and 100 U/mL Penicillin – 100 µg/mL Streptomycin (Fisher Scientific, #11548876)]. All cells were maintained at 37 °C, 5% CO_2,_ and passaged at ~ 95% confluency. For passaging, cells were first washed with 1X DPBS (Fisher Scientific, #10444402) pre-warmed to 37 °C, then detached from the dish surface with TrypLE^TM^ (Fisher Scientific, #12604021), resuspended in complete medium, and counted using a hemocytometer (THOMA chamber, Carl Roth, #T732.1) in the presence of 0.02% Trypan Blue Stain (Thermo Fisher, #15250061) to assess cell viability.

For telomerase activity assays, one million WI-38 VA13 cells were seeded into a 100 mm dish. After incubation for 24 h, the media was aspirated, and the cells were washed once with Opti-MEM (Fisher Scientific, #15392402) pre-warmed to 37 °C. The media was replaced with 10 mL of pre-warmed Opti-MEM, and the cells were returned to the incubator during transfection cocktail preparation. For each dish, the following reagents were mixed in a 1.5 mL tube: 1 µg pcDNA6-3HA-hTERT plasmid, 5 µg of either pBS-U1-WT-hTR, pBS-U1-C50>A-hTR generated by site-directed mutagenesis, or a 1:1 mix of both plasmids, 18 µL of FuGENE HD transfection reagent (Promega, #E2311), and Opti-MEM media to a final volume of 750 µL. The transfection cocktail was incubated at room temperature for 20 min before drop-wise addition to the cell culture dish. Four hours later, 2 mL of FBS (LGC standards, #ATCC-30-2020) pre-warmed to 37 °C was added to each dish. After 48 h, the media was replaced with complete medium and 24 h later, the cells were detached with TrypLE^TM^, pelleted at RT by centrifugation at 200 × *g* for 5 min, and CHAPS extracts were prepared.

### CHAPS Extract preparation

Cell pellets were thoroughly re-suspended and lysed by pipetting in one packed cell volume of CHAPS buffer [0.5% w/v CHAPS (Merck, #C5070), 50 mM Tris-HCl at pH 7.5, 50 mM KCl, 1 mM MgCl_2_, 1 mM EGTA at pH 8, 10% (v/v) glycerol, 5 mM DTT, 1 mM PMSF (stock solution: 100 mM in isopropanol)]. Following rotation at 10 rpm at 4 °C for 1 h, the lysates were cleared by centrifugation at 16,000 × *g* (Eppendorf, #Centrifuge 5430 R) at 4 °C for 20 min. Supernatants were transferred into fresh 1.5 mL tubes, snap-frozen in liquid nitrogen, and stored at − 80 °C. An aliquot of each lysate was used to measure the protein concentration by Bradford assay.

### Bradford assay

CHAPS extract was diluted 1:5 with CHAPS buffer, and 1 µL was transferred to a fresh 1.5 mL tube. 5 µL of ddH_2_O and 994 µL of Bradford reagent (Bio-Rad, #5000205) were added to the extract and incubated at RT for 5 min, before measuring the sample absorbance at 595 nm with a UV-Vis spectrophotometer (Amersham Biosciences, Ultrospec 2100 pro). The protein concentration of the sample was calculated based on a standard curve obtained with BSA (Bio-Rad, #5000201) diluted in 5 µL ddH_2_O, 1 µL of CHAPS buffer and 994 µL of Bradford reagent.

*Western Blotting* CHAPS extracts (5 µL at 1 µg/µL) was diluted 1:1 with 2X SDS loading buffer [4X LDS Sample Buffer (Fisher Scientific, #11549166), 100 mM DTT (Fisher Scientific, #10386833), 4% SDS (Carl Roth, #8029.3)] and heated at 95 °C for 5 minutes. Samples were loaded onto a 4 − 12% polyacrylamide gradient gel (Fisher Scientific, #10338442) and run for at least 1 h at 150 V in 1X MOPS buffer (Fisher Scientific, #11589156) at RT. Proteinswere transferred to nitrocellulose membrane for 1 h at 100 V at 4 °C in 1X Transfer Buffer [25 mM Tris Base (Fisher Scientific, #10376743), 192 mM Glycine (Fisher Scientific, #10070150), 20% Methanol V/V (Carl Roth, #0082.3), pH = 8.3]. The membrane was blocked for 1 h at RT with gentle shaking in Blocking solution [5% Milk powder (Carl Roth, #T145.2) in 1X TBST (20 mM Tris-Base (Fisher Scientific, #10376743), 150 mM NaCl (Merck, #S9888), 0.1% V/V Tween™ 20 (Fisher Scientific, #10113103), pH = 7,6]. The blocked membrane was incubated overnight at 4 °C with gentle shaking with primary antibodies mouse α-HA-Tag (Invitrogen, #261831MG, Lot: YB370961, 1:1000 diluted) and mouse α-Tubulin (Merck, #T5168 Batch: 0000126956, 1:4000 diluted) diluted in Blocking solution. Subsequently, the membrane was washed three times with 1X TBST at RT for 5 min with gentle shaking, before incubation for 1 h at RT with gentle shaking with goat α-Mouse-HRP conjugated secondary antibody (Invitrogen, #31430, 1:10000, Lot: 3408712) diluted in Blocking solution. Finally, the membrane was washed again three times with 1X TBST at RT for 5 min with gentle shaking, before developing of the signal with Clarity Max Western ECL Substrate (Bio-Rad, #1705062). The developed membrane was then imaged with ChemiDoc™ MP (Bio-Rad, #17001402), and the resulting images were analyzed with the Image Lab software (Bio-Rad).

### Telo-FISH

HeLa cells were grown in EMEM (Fisher Scientific, #11440335), 10% fetal bovine serum (LGC standards, #ATCC-30-2020), and 2 mM L-Glutamine (Fisher Scientific, #25030081) at 37 °C, 5% CO_2,_ and 20% O_2_. 5 × 10^5^ cells were seeded onto 22 ×22 mm high-precision microscope cover glasses in 6-well TC plates and grown for 96 h to a confluency of 75–80%. Cells were fixed for 10 min in 2 mL of mixing buffer [50% (v/v) MeOH, 50% (v/v) EtOH] for 10 min, 5 min in 2 mL 4% paraformaldehyde in PBS, and washed three times for two minutes in 1X PBS (2 mL). Cells were incubated with 100 µg/mL RNase A (Fisher Scientific, #10753721) in 2X SSC at 37 °C for 60 min in a humidity chamber and dehydrated in a graded series of ethanol (70, 80, and 100%) for 5 min each. Slides were air-dried for 5 min. Cells were incubated in a 100 nM solution of Alexa 647-labeled telomeric PNA probe [PNA Bio, #F1014; (TTAGGG)_3_] and 100 nM of Alexa 488-labeled mutant telomere PNA probe [PNA Bio (GGTTTA)_3_ in hybridization solution, containing 70% ultrapure formamide, 0.1 µg/mL salmon sperm DNA solution (Invitrogen, #15632011), and 10 mM Tris-HCl (pH 7.2) for 3 minutes at 85 °C in an UVP Hybridizer Oven (Analytik Jena) followed by overnight incubation in the dark at room temperature. Slides were washed twice for 15 min in 2 mL 70% formamide/10 mM Tris-HCl (pH 7.2), followed by three washes for 5 min in 100 mM Tris-HCl/150 mM NaCl/0.08% Tween-20 (pH 7.5). Slides were incubated in 1:1000 (2 mg/mL) DAPI in 2X SSC for 5 min and mounted in Prolong Glass Antifade Mountant (Fisher Scientific, #1589391).

### Direct telomerase activity assay (DTAA)

DTAAs were assembled in 20 µL reactions containing CHAPS extract in quantities indicated in each figure, 1X reaction buffer [50 mM Tris–HCl (pH 8.0), 50 mM KCl, 1 mM spermidine (Merck, #S0266), 1 mM β-mercaptoethanol (Merck, #M3148), 1 mM MgCl_2_], 1 mM dATP, 1 mM dTTP, 5.83 µM dGTP (10 µCi of [α−32P]dGTP at 3000 Ci/mmol, Perkin Elmer, #NEG514H), and 0.7 µM of the telomeric primer. Reactions were incubated for 30 min at 30 °C and stopped by the addition of 5 µL of 25 mM EDTA (Fisher Scientific, #10618973) and 5% SDS (Carl Roth, #8029.3). A ^32^P-labeled 14-mer DNA oligonucleotide (1000 counts per minute) was added as a recovery control to each sample. Products were ethanol-precipitated overnight at − 20 °C with 2.5 volumes of 100% ice-cold ethanol and 0.25 volumes of 10 M ammonium acetate. DNA pellets were re-suspended in 10 µL of 2X RNA Loading Dye (NEB, #B0363S) and heated to 95 °C for 5 min. Samples (5 µL) were then loaded onto a pre-run (1 h at 80 W in 1 x TBE) denaturing gel [12% polyacrylamide 19:1 (Fisher Scientific, #10214963), 8 M urea (Merck, #U5378)] in 1X TBE [Tris/Borate/EDTA buffer: 0.1 M Tris Base (Fisher Scientific, #10376743), 0.1 M Boric Acid (Merck, #B6768), 2 mM EDTA (Fisher Scientific, #10618973)]. The samples were subjected to electrophoresis at 80 W for 100 min. Gels were vacuum dried between a plastic film (VWR, #LONZ54746) and a sheet of Whatman paper (Fisher Scientific, #11330744) at 60 °C for 45 minutes. Finally, the gel was exposed overnight to a phosphor screen in a cassette and imaged with a Typhoon Scanner (cytiva, GE healthcare, #29193226).

### DTAA Quantification

Images were quantified using ImageJ software v1.52i^51^, adapting the method used by Latrick & Cech^52^. For all lanes, the counts for each visible extension product were obtained individually, normalized for the number of radiolabeled guanosines contained within, normalized against the loading control, and then summed over the entire lane to obtain the total lane counts (TLCs). TLCs were further normalized to the TLC of the wild-type sample and used as a metric of relative telomerase engagement. For processivity calculations, given an extension product of size n, the percentage left behind (%LB) was calculated by adding the sum of counts for the bands corresponding to smaller products than band n to the counts for band n itself, divided by the TLC, and multiplied by 100. The ln(100 - %LB) was plotted over the number of nucleotides added to the substrate primer. The data points for each lane were fit with a linear regression equation, where the processivity value equals ln(2)/slope of regression.

### RNA Isolation from CHAPS Extract

CHAPS extracts (500 µg in 25 µL) was mixed with ddH_2_O (75 µL) and 25 µL of 5X Stop Buffer [100 mM Tris-HCl at pH 7.5, 200 mM EDTA (Fisher Scientific, #10618973), 10 mg/mL proteinase K (Merck, #P2308), 2.5% w/v Sodium dodecyl sulfate (SDS, Carl Roth, #8029.3)] followed by incubation at 50 °C at 500 rpm in an Eppendorf ThermoMixer F1.5 for 15 min. An additional 175 µL of ddH_2_O was added to the reaction, followed by RNA extraction with an equal volume of Phenol:Chloroform:Isoamyl Alcohol (300 µL, 25:24:1, pre-equilibrated with 50 mM NaOAc pH 5.2, Merck, #P2069). The RNA sample was ethanol precipitated overnight at − 20 °C with 2.5 volumes of ice-cold 100% Ethanol, 1/10 volume of 3 M NaOAc pH 5.2 (Merck, #W302406), and 1 µL of Glycogen (5 mg/mL, Fisher Scientific, #AM9510). The RNA pellets were washed with 1 mL of ice-cold 70% ethanol, air-dried, and suspended in 168 µL of ddH_2_O + 2 µL of RNasin Plus (Promega, #N2615). DNase treatment was performed at 37 °C for 1 h following the addition of 20 µL of TURBO DNase Buffer 10X and 10 µL of TURBO DNase (2 µ/µL, Fisher Scientific, #10722687). Subsequently, 100 µL of ddH_2_O was added to the sample before Phenol:Chloroform:Isoamyl Alcohol extraction and ethanol precipitation for 4 h at − 20 °C as described above. The precipitated RNA was air-dried and dissolved in 88 µL of ddH_2_O and 1 µL of RNasin Plus. A second DNase treatment was performed by the addition of 10 µL of TURBO DNase Buffer 10X and 1 µL of TURBO DNase, incubating the sample at 37 °C for 30 min. The second DNase treatment was necessary to completely remove the plasmid DNA present in the extract made from super-telomerase transfected cells. Finally, 200 µL of ddH_2_O was added to the sample before a final Phenol:Chloroform:Isoamyl Alcohol RNA extraction and ethanol precipitation overnight at − 20 °C as described above. The precipitated RNA was air-dried and dissolved in 16 µL of ddH_2_O. 1 µL of sample was used for NanoDrop (Thermo Fisher Scientific, #ND-ONE-W) quantification, before snap-freezing in liquid nitrogen and storage at − 80 °C.

### Quantitative PCR

RNA samples (1 µg) were reverse transcribed in 20 µL reactions using the InvitrogenTM SuperScript™ VILO™ cDNA-Synthesis kit (Fisher Scientific, #11754250). The thermo-cycler settings were as follows: 25 °C for 10 minutes; 42 °C for 1 h; 85 °C for 5 min; hold at 4 °C. After the reverse transcription, 1 µL of RNase H (NEB, #M0297) was added, and reactions were incubated at 37 °C for 20 min. RNase H was heat-inactivated at 65 °C for 20 minutes. Quantitative real-time PCR was performed in a 10 µL reaction containing 2 µL of cDNA sample diluted 1:1 with ddH_2_O, 1 µL of primer mix (final concentrations: 500 nM forward and 500 nM reverse primer), 5 µL FastMix™ with Low-ROX reference dye 2X (VWR, #733-1390), and 2 µL of ddH_2_O. A list of all the primers used in this manuscript can be found in Supplementary Table 3. The samples were transferred into a 384-well plate (Fisher Scientific, #10005724) and sealed with an adhesive film (Fisher Scientific, #10299204). The reaction was performed on a ViiA 7 Real-Time PCR System, and the results were analyzed with QuantStudio Real-Time PCR Software v1.3. The machine settings were as follows: 50 °C for 2 min, 95 °C for 10 min, 40 cycles of 95 °C for 15 s, 60 °C for 1 min, 95 °C for 15 s, 60 °C for 1 min, and 95 °C for 15 s. The temperature was altered at a speed of 1.6 °C/s at all stages except for the final change between 60 °C and 95 °C, where the speed was 0.05 °C/s to determine the melting curve for each primer. The fluorescence signal was quantified during the first hold at 60 °C.

### Telomere length determination

Telomere length measurements were carried out as described^53^. Briefly, NH_4_Cl was used for erythrocyte lysis in peripheral blood. Leukocytes were mixed with bovine thymocytes as an internal control with known telomere length. Cells were then denatured in formamide at 87 °C and hybridized with a telomere-specific fluorescein-conjugated (CCCTAA)_3_ peptide nucleic acid (PNA) probe and counterstained with LDS751 DNA dye. The fluorescence intensity of the telomere signal in granulocyte and lymphocyte subsets defined by specific antibodies for CD20 (CD20 eFlour 615, Clone HI100, Biolegend #304130, Lot #B359105, 18 µg/ml), CD45RA (CD45RA BV 421, Clone L25, eBioscience #42-0202-82, Lot #2567403, 135 µg/ml), and CD57 (CD57 PE, Clone NK-1, BD Pharmingen #560844, Lot #2245781, 2.25 µg/ml) was measured on a FACSCanto (Becton Dickinson) and was set in relation to the internal bovine thymocytes and unstained control cells.

### Nucleic acid extraction and sequencing

Whole genome sequencing (WGS) was conducted with DNA isolated from cultured fibroblasts and whole blood, respectively. DNA was isolated from cultured fibroblasts at passage 3 by suspending the cell pellets in 200 µL SE-Buffer (75 mM NaCl, 25 mM Na-EDTA), 20 µL 10% SDS, and 20 µL Proteinase K (15 mg/mL). After overnight incubation at 37 °C, 80 µL 6 M NaCl was added and mixed by vortexing, followed by centrifugation at 6000 × *g* for 15 min, and recovery of the supernatant. For DNA precipitation 100% ethanol (600 µL) was added to the supernatant and centrifuged at 14,000 × *g* for 20 min. The DNA pellet was washed with 1 mL 70% Ethanol. The wash process was repeated once prior to air drying and the solubilization of the pellet in 20 µL H_2_O. DNA was isolated from whole blood using Chemagen Chemagic DNA Blood Kit Special for 5 mL and the Chemagen Robot MSM1 following the manufacturer’s instructions. WGS libraries were prepared using NEBNext^®^ Ultra^™^ II FS DNA Library Prep Kit for Illumina with enzymatic fragmentation and sequenced to a minimum depth of 250 million read pairs (2x150nts) on an Illumina NextSeq2000. Reads were trimmed using Trimmomatic^54,55^ PE v0.39 with the following parameters: SLIDINGWINDOW:5:15, LEADING:10, TRAILING:10, MINLEN:100, and ILLUMINACLIP:TruSeq3-PE.fa:2:20:10. Alignment to human reference GRCh38 was performed using BWA MEM^56^ v0.7.17. Read counts, quality distributions, and alignment results can be found in Supplementary Table 4.

For RNA extraction, whole blood was collected and stored in PAXgene Blood RNA Tubes (PreAnalytiX) at − 20 °C prior to further processing. Samples were brought to room temperature for 4 h, and total RNA was extracted using the PAXgene Blood RNA Kit (Qiagen Cat. No. 762174) and manufacturer’s recommendations (April 2022, v3). Libraries were prepared using Illumina’s Stranded Total RNA Prep Ligation with Ribo-Zero Plus Kit following Stranded Total RNA prep Ligation with Ribo-Zero Plus Reference Guide (August 2020, v01), starting with 1000 ng and 9 PCR cycles of 98 °C for 10 s, 60 °C for 30 s, 72 °C for 30 s, followed by a 72 °C hold for 5 min. Libraries were sequenced to a minimum depth of 195 million read pairs (2x75nts) on an Illumina NextSeq500 Midoutput FC. Reads were trimmed using Trimmomatic PE^54^ v0.39 with the following parameters SLIDINGWINDOW:5:10, LEADING:5, TRAILING:5, MINLEN:18, and ILLUMINACLIP:TruSeq3-PE.fa:2:20:10. Trimmed reads were aligned to human reference GRCh38 using STAR^57^ v2.7.10b and default parameters. Read counts, quality distributions, and alignment results can be found in Supplementary Table 5.

### Identification of telomeric sequences

Telomeric sequences were isolated from unprocessed WGS data obtained from the DNA extractions described in the section above. Telomeric reads were first isolated based on the combined presence of 7 GGTTAG or GTTTA repeats or the respective reverse complements, CTAACC and TAAAC. Isolated reads were trimmed using Trimmomatic^54^ PE v0.39 with a SLIDINGWINDOW parameter of 5:15 to remove low-quality bases. Reads were then trimmed to the first and last presence of a wild-type or mutant telomeric repeat. Fully telomeric reads were classified as having a length greater than 80 nucleotides and containing more telomeric repeats (wild-type or mutant) than the length of the read divided by six minus two. Fully telomeric C-strand sequences were then reverse-complemented for further analysis. The mutant ratio is defined as the number of bases contributing to mutant repeats normalized to the number of bases in fully telomeric reads, while the wild-type ratio is the number of bases contributing to wild-type telomeric repeats normalized for the number of bases in fully telomeric reads. Wild-type ratio plus mutant ratio equals less than one: the remaining fraction is biological and technical noise in the dataset contributed by insertions and non-wild-type telomeric repeats or sequences. Read counts and telomere related statistics are found in Supplementary Table 6.

### Nanopore sequencing of telomeres

gDNA was extracted from whole blood using the Qiagen Midi Kit according to the manufacturer’s recommendations or via the robotic DNA extraction method previously described. Telomeres were enriched prior to sequencing using the duplex enrichment protocol previously described^24,25^ with 3.75 µL of 100 nM capture probe duplex. An equimolar combination of capture probes (Supplementary Table 3, BLoli9187-BLoli9194 duplexed with BLoli9202) was used to ensure that all possible combinations of wild-type and mutant telomeric repeats would be captured. Sequencing was performed on a R10.4.1 MinION flowcell for 72 h and data was basecalled using Dorado v0.7.0 and super high accuracy basecalling model v5.0.0 prior to analysis with TARPON^25^ using default parameters to extract telomeric sequences. Read counts and telomere related statistics are found in Supplementary Table 7.

### Telomerase processivity analysis

WGS Illumina and telomere-enriched nanopore long read sequencing data was used to calculate in vivo processivity for mutant and wild-type telomerase. As mutant and wild-type hTR are present in equal proportions and under the assumption that they have an equal likelihood of associating with telomeres, as the annealing region of hTR is unaffected, the *p*(association) of each enzyme is 0.5. An additional mutant repeat can originate either by processive extension by the same enzyme, *p*(processive), or by disassociation and re-association of another mutant telomerase, (1 – *p*(processive)) * *p*(association). Since these two events are independent, their summation equals the probability that the next telomeric repeat will also be a mutant repeat, *p*(consecutive) (*eq1*). By plotting the length of repeat tracts against the natural log of the frequency of each given length, the resultant slope can be used to calculate *p*(consecutive) and therefore *p*(processive). Mutant repeat tracts were identified by isolating sequences greater than 4 nucleotides between two wild-type repeats and included for analysis if the isolated nucleotides were composed of greater than 60% mutant telomeric repeats. Wild-type repeat tracts were identified by isolating sequences between two mutant repeats and included in the analysis if the resulting sequence was composed solely of wild-type repeats. Stretches of consecutive repeats for Nanopore sequencing were only identified within the last 1 kb of the read to ensure telomerase active regions were being used to calculate processivity. To remove outliers that skew processivity calculations, data for each wild-type or mutant repeats was fitted to a negative binomial distribution by maximum likelihood estimation. The distribution’s 99.9^th^ percentile was calculated, and any larger repeat stretch was removed.

1 $$p({consecutive})=p({processive})+(1-p({processive}))*p({association})$$

### Reporting summary

Further information on research design is available in the Nature Portfolio Reporting Summary linked to this article.

## Supplementary information

Supplementary Information

Reporting Summary

Transparent Peer Review file

## Source data

Source Data

## Data Availability

The raw sequencing data supporting the findings of this study have been deposited in the European Genome-phenome Archive (EGA), which is hosted by the EBI and CRG. The data supporting the findings of this study contain sensitive human participant information and cannot be made publicly available due to ethical and privacy restrictions. The data is available under restricted access via dataset accession EGAS50000001644[https://ega-archive.org/dacs/EGAC50000000925]. Data can be accessed for research purposes by authorized individuals. Further details on how to access the data, including the Data Access Committee (DAC) policy and Data Access Agreement requirements, can be found directly on the dataset page at https://ega-archive.org/“. Source data are provided in this paper.

## Code availability

All custom code used for the analysis of sequencing data is publicly available at https://github.com/baumannlab/DC_Telomere_Analysis_2026 under release DC_2026 10.5072/zenodo.569893
