# Supplementary material for: RTEL1 mutation modifies dyskeratosis congenita caused by a telomerase RNA template mutation
Source: Nat Commun. 2026 Aug 26;17:9054. doi: 10.1038/s41467-026-76650-w (PMC13518968; doi:10.1038/s41467-026-76650-w)
Supplement: Supplementary file 1 [file 41467_2026_76650_MOESM1_ESM.pdf]

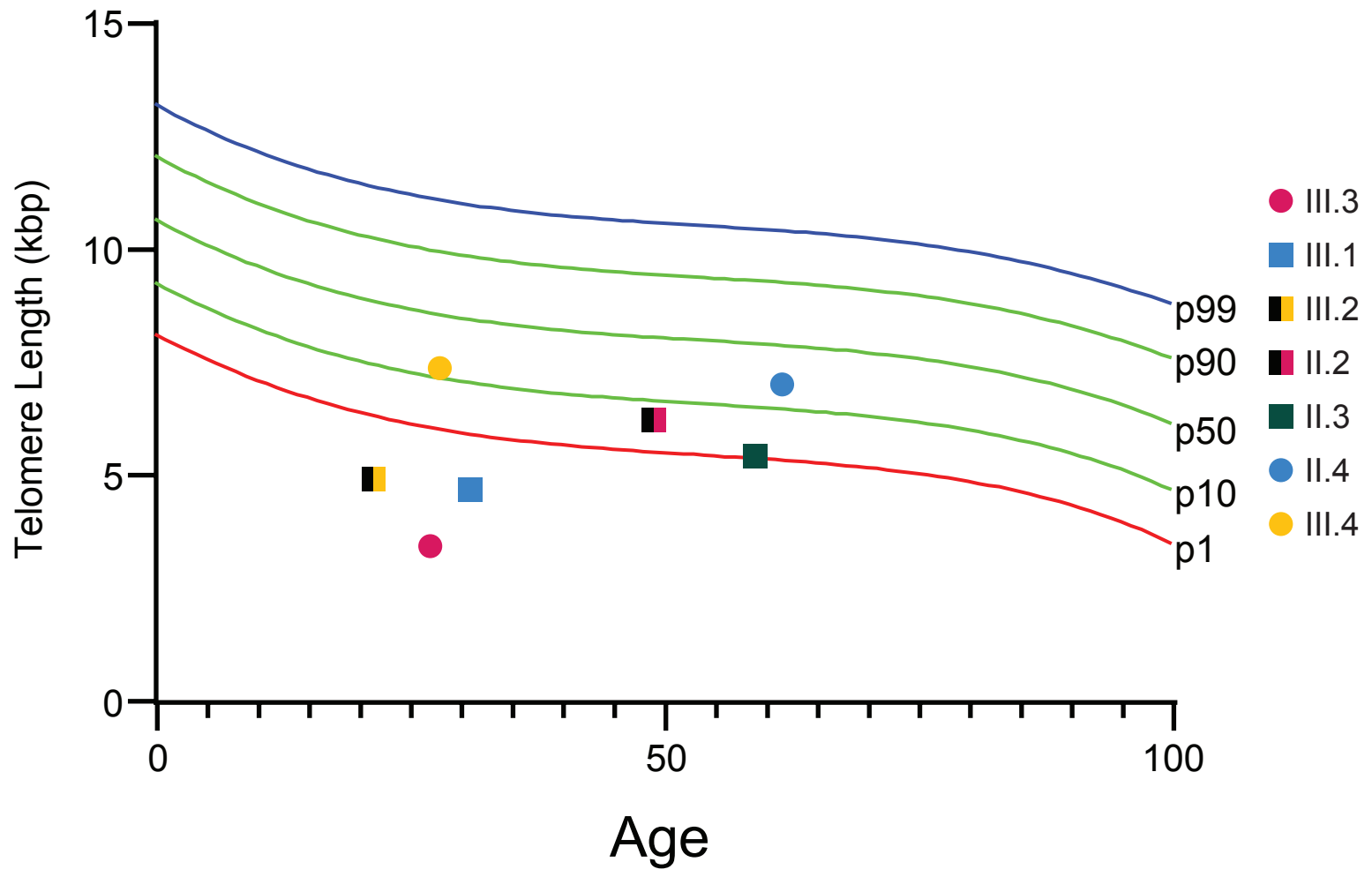

Supplemental Figure 1. Granulocyte flow-FISH measured telomere length for all individuals in this study excluding II.1 where no data is available. Individuals harboring the RTEL1 nonsense mutation (III.1, III.2, and III.3) all show telomeres lengths below the 1st percentile in granulocytes, while those harboring only the C50>A mutation have telomeres shorter than the tenth percentile. Half shaded squares represent deceased individuals. Individuals with neither mutation or inherited mutant telomeric repeats show telomere lengths greater than the tenth percentile.

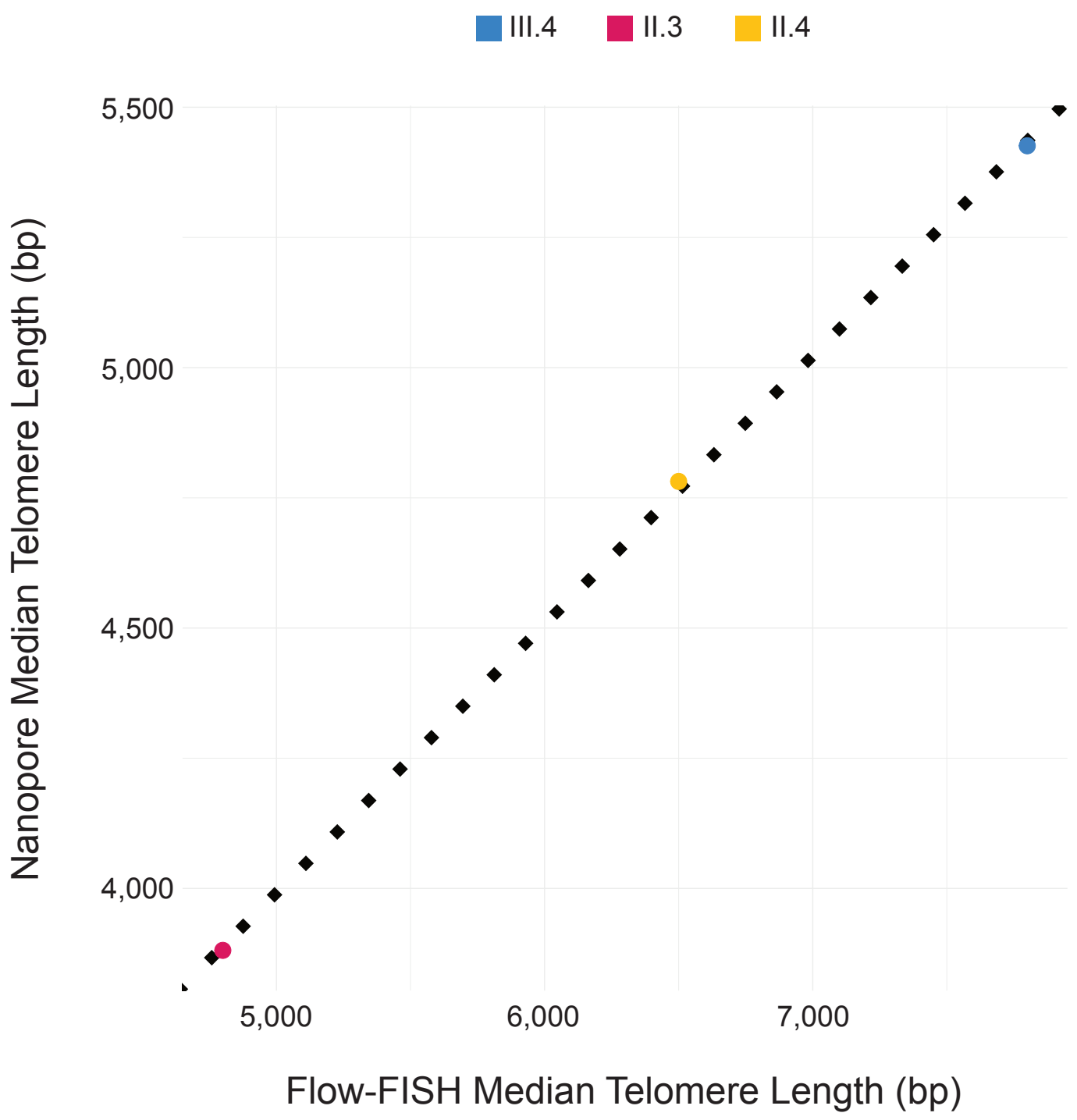

Supplemental Figure 2. Correlation between Nanopore calculated median telomere length and Flow-FISH median telomere lengths shows a strong correlation. R2=0.9993, p-value=0.01181.

a

b

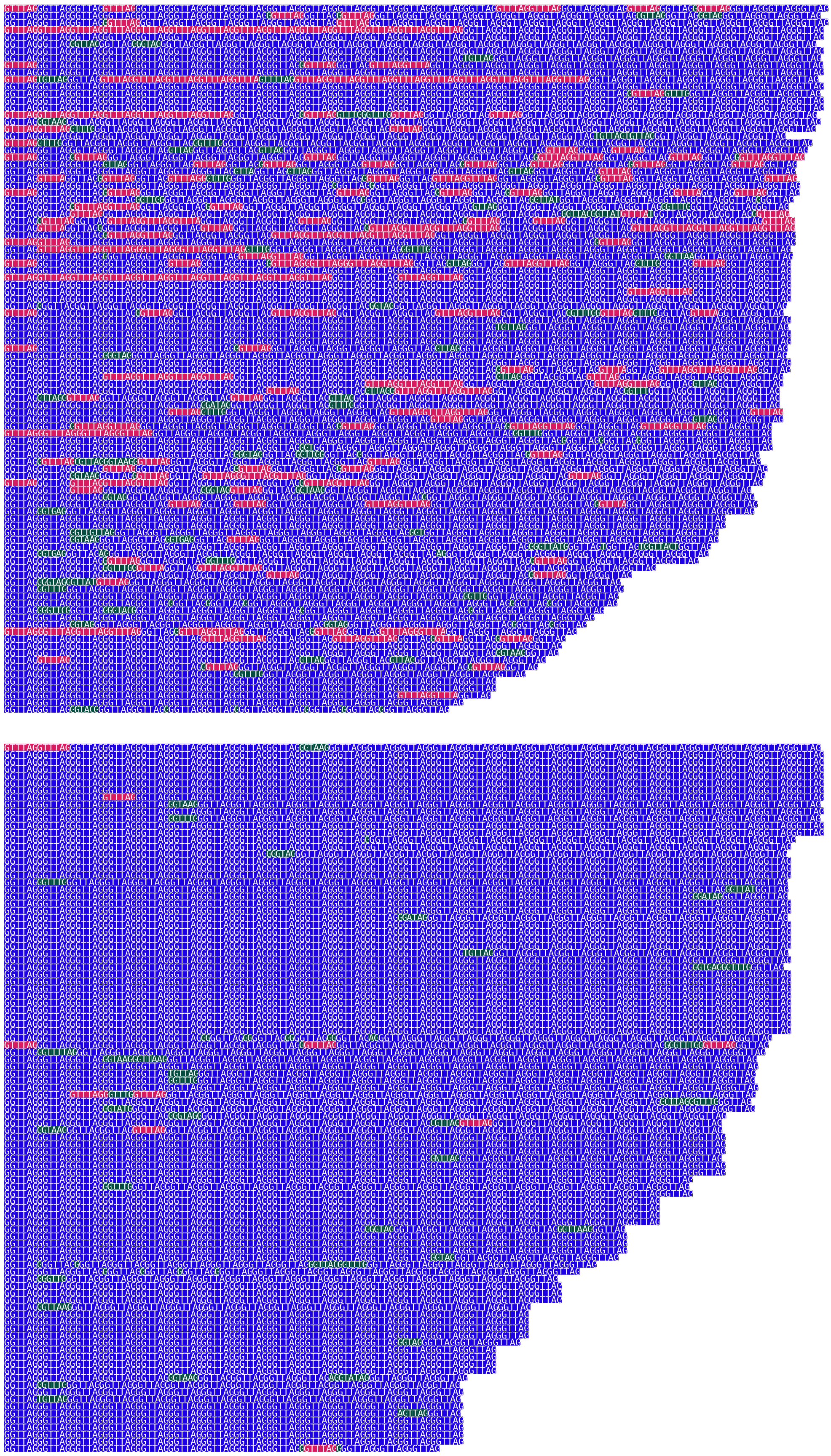

Supplemental Figure 3. (a) 100 randomly selected telomeric sequences for III.3 or **(b)** an age matched control with wild-type telomeric repeats highlighted in blue, GTTTAG mutant repeats in red, and all other sequences in green demonstrating the biological and technical noise within the dataset and the increased frequency of GTTTA mutant telomeric sequence in the proband.

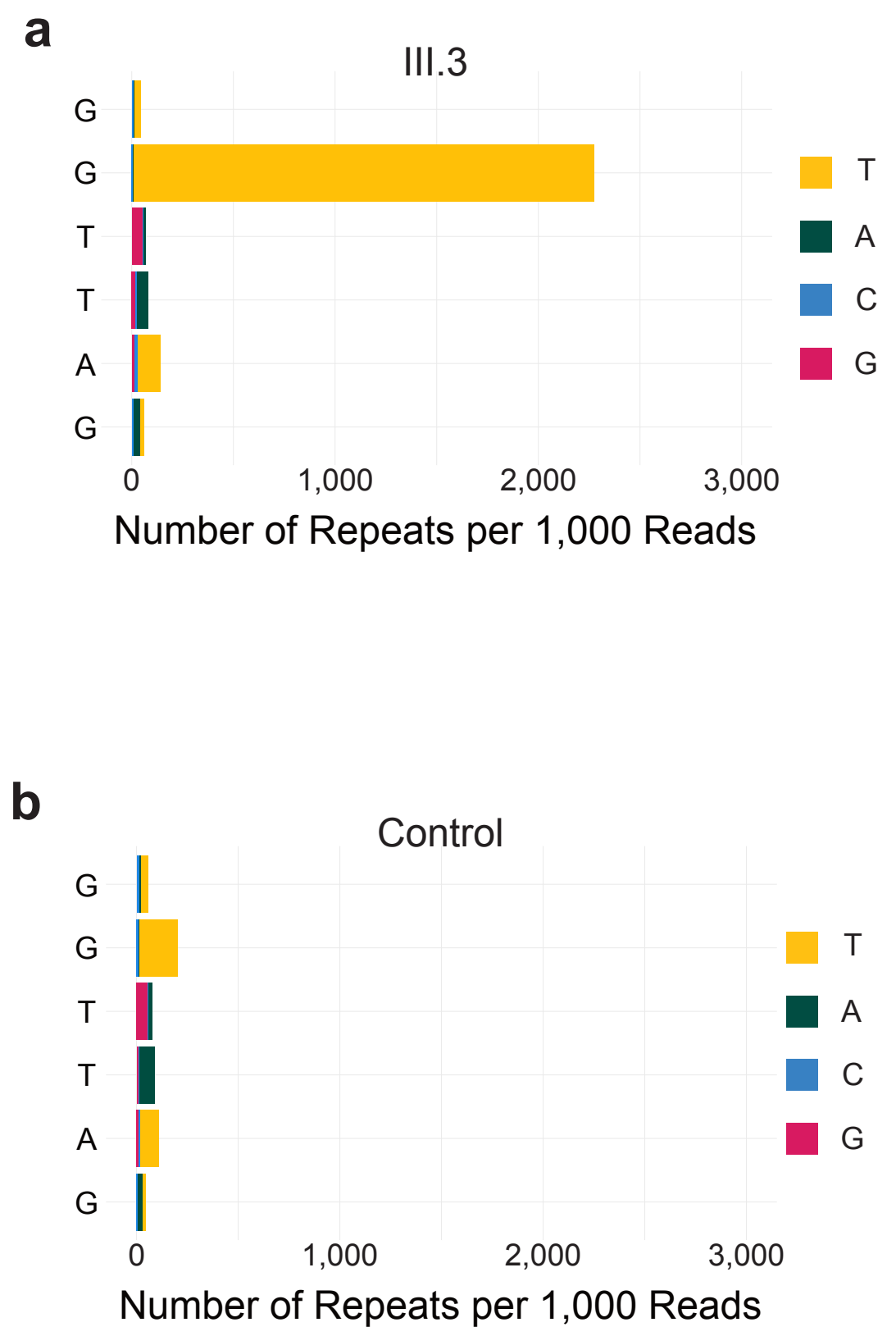

Supplemental Figure 4. Distribution of one nucleotide point mutations of the wild-type telomeric repeat across telomeric sequences isolated from Illumina WGS data from blood of **(a)** C50>A hTR mutant containing individual III.3 and **(b)** a control

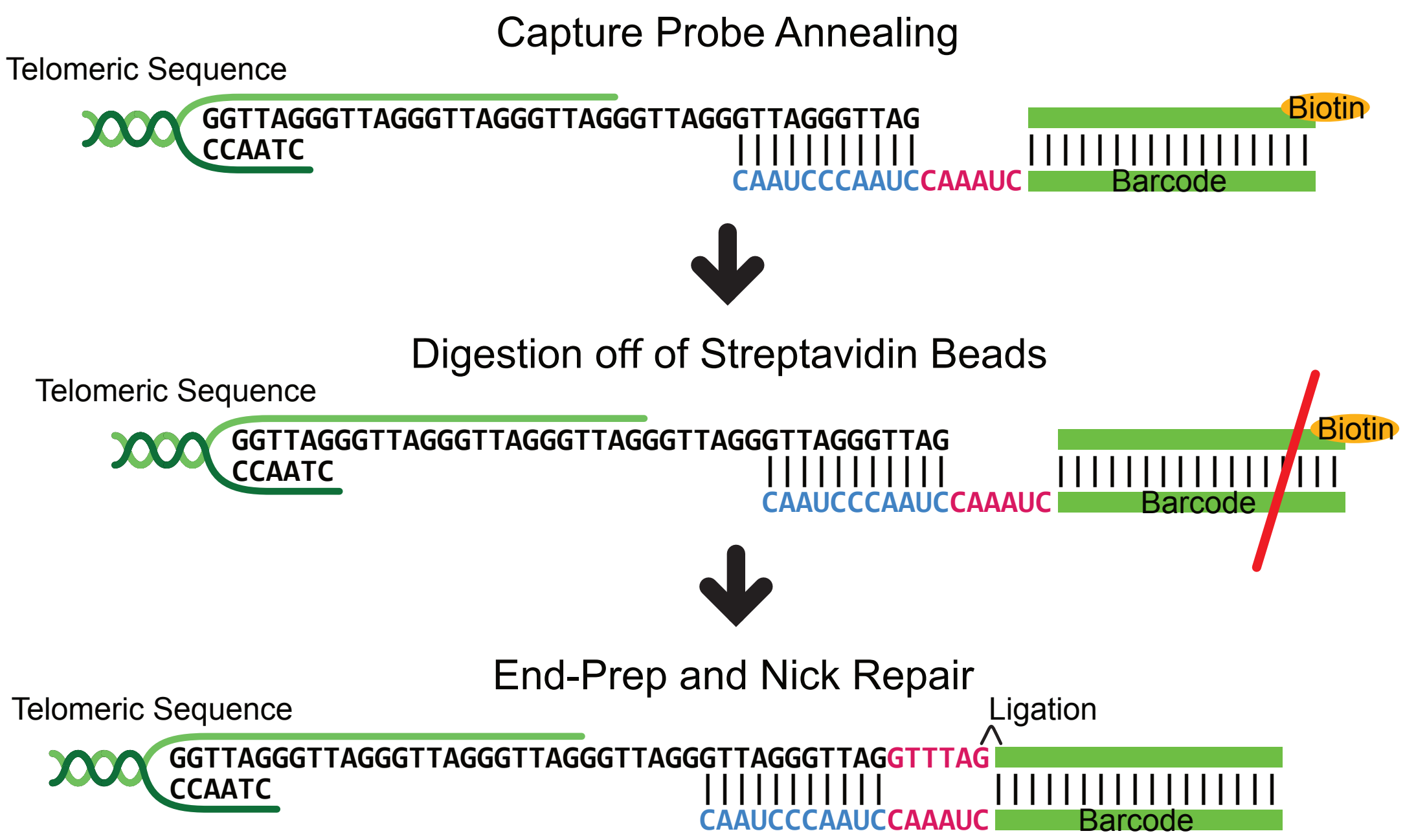

Supplemental Figure 5. Schematic of sequencing artifact identified in Nanopore sequencing data sets. The capture probe anneals to the telomeric 3′ overhang leaving a gap. A terminal mutant telomeric repeat is then copied to the end of a wild type telomeric sequence by the End-Preparation and Nick Repair step of Nanopore library preparation.

Supplemental Table 1. Illumina WGS and ribodepleted RNA sequencing coverage of all patients at the TERC r.C50>A locus.

				DNA			RNA		
**Patient**	**Relationship to Index Patient**	**Tissue**	**Status**	**Coverage**	**WT**	**MT**	**Coverage**	**WT**	**MT**
III.3	-	Blood	Heterozygous	21	9 (43%)	12 (57%)	534	258 (48%)	276 (52%)
III.3	-	Fibroblasts	Heterozygous	10	7 (70%)	3 (30%)	N/A		
II.3	Aunt	Blood	Heterozygous	11	7 (64%)	4 (36%)	220	116 (53%)	104 (47%)
III.2	Twin Sister	Fibroblasts	Heterozygous	28	13 (46%)	15(54%)	N/A		
III.1	Older Sister	Blood	Homozygous WT	12	12 (100%)	0 (0%)	201	201 (100%)	0 (0%)
II.2	Mother	Blood	Heterozygous	23	12 (52%)	11 (48%)	N/A		
III.4	Cousin	Blood	Homozygous WT	24	23 (96%)	1 (4%)*	N/A		
III.2	Twin Sister*	Blood	Homozygous WT	23	23 (100%)	0 (0%)	N/A		
Control	C_0079	Blood	Homozygous WT	16	16 (100%)	0 (0%)	N/A		
Control	C_0079	Fibroblasts	Homozygous WT	20	20 (100%)	0 (0%)	N/A		
Control	C_1262	Blood	Homozygous WT	13	13 (100%)	0 (0%)	N/A		
Control	C_1262	Fibroblasts	Homozygous WT	18	17 (94%)	1 (6%)*	N/A		

*Post bone marrow transplant - Data represents individual II.1; the father of the index Patient

Supplemental Table 2. Illumina WGS and ribodepleted RNA sequencing coverage at the location of the Q1178x RTEL1 mutation on Chromosome 20:63,695,360 (C>T), GRCh38 primary assembly.

				DNA			RNA		
Patient	Relationship to Index Patient	Tissue	Status	Coverage	WT	MT	Coverage	WT	MT
III.3	-		Heterozygous	16	8 (50%)	8 (50%)	28	19 (68%)	9 (32%)
III.3	-		Heterozygous	23	16 (70%)	7 (30%)	N/A		
III.3	Aunt	Blood	Homozygous WT	18	18 (100%)	0 (0%)	16	16 (100%)	0 (0%)
III.2	Twin Sister	Fibroblasts	Heterozygous	41	24 (59%)	17 (41%)	N/A		
III.1	Older Sister	Blood	Heterozygous	9	1 (11%)	8 (89%)	21	16 (76%)	5 (24%)
II.2	Mother	Blood	Homozygous WT	21	21 (100%)	0 (0%)	N/A		
III.4	Maternal Cousin	Blood	Homozygous WT	44	44 (100%)	0 (0%)	N/A		
III.2	Twin Sister*	Blood	Heterozygous	35	23 (66%)	12 (34%)	N/A		
Control	C_0079	Blood	Homozygous WT	14	14 (100%)	0 (0%)	N/A		
Control	C_0079	Fibroblasts	Homozygous WT	19	19 (100%)	0 (0%)	N/A		
Control	C_1262	Blood	Homozygous WT	14	14 (100%)	0 (0%)	N/A		
Control	C_1262	Fibroblasts	Homozygous WT	17	17 (100%)	0 (0%)	N/A		

*Post bone marrow transplant - Data represents individual II.1; the father of the index Patient

Supplemental Table 3. Oligonucleotide and primer sequences used in this study. Oligonucleotides BLoli9187 to Bloli9194 were pooled with BLoli9202 and annealed to create a capture probe mixture of duplexes.

Name	Gene	Sequence (5'>3')	Use
BLoli5319	ATP5β Forward primer	GATCCTCTAGACTCCACCTCTC	Endogenous control for qPCR
BLoli5320	ATP5β Reverse primer	AGAAAGTTCATCCATACCCAGG	Endogenous control for qPCR
BLoli5327	GAPDH Forward primer	ACATCGCTCAGACACCATG	Endogenous control for qPCR
BLoli5328	GAPDH Reverse primer	TGTAGTTGAGGTCAATGAAGGG	Endogenous control for qPCR
BLoli5317	HPRT Forward primer	TGCTGAGGATTTGGAAAGGG	Endogenous control for qPCR
BLoli5318	HPRT Reverse primer	ACAGAGGGCTACAATGTGATG	Endogenous control for qPCR
BLoli8342	TERT mRNA Forward primer	GCAAGTTGCAAAGCATTGGA	Verifying TERT overexpression by qPCR
BLoli8343	TERT mRNA Reverse primer	ACCTCTGCTTCCGACAGCTC	Verifying TERT overexpression by qPCR
BLoli3834	Total-hTR Forward primer	GCGAAGAGTTGGGCTCTGTCA	Verifying hTR overexpression by qPCR
BLoli3835	Total-hTR Reverse primer	TTCCTCTTCCTGCGGCCTGAAA	Verifying hTR overexpression by qPCR
BLoli8868	RTEL1 mRNA Forward primer	TTCTCACAGACGTGCACAGA	Measuring RTEL1 expression levels by qPCR
BLoli8869	RTEL1 mRNA Reverse primer	CATCCTAGAGGCCCCACTCA	Measuring RTEL1 expression levels by qPCR
BLoli9187		GAGTCCACCACGATCGTGAATTGCTGTACGCAACGCAAG TCAACCATGAGCGCAATATATCGGAGGGCTAACCCTAAC CCTAACC	WT-WT-WT Duplex Capture Probe Overhang
BLoli9188		GAGTCCACCACGATCGTGAATTGCTGTACGCAACGCAAG TCAACCATGAGCGCAATATATCGGAGGGCTAACCCTAAC CCTAAAC	WT-WT-MT Duplex Capture Probe Overhang
BLoli9189		GAGTCCACCACGATCGTGAATTGCTGTACGCAACGCAAG TCAACCATGAGCGCAATATATCGGAGGGCTAACCCTAAA CCTAACC	WT-MT-WT Duplex Capture Probe Overhang
BLoli9190		GAGTCCACCACGATCGTGAATTGCTGTACGCAACGCAAG TCAACCATGAGCGCAATATATCGGAGGGCTAAACCTAAC CCTAACC	MT-WT-WT Duplex Capture Probe Overhang
BLoli9191		GAGTCCACCACGATCGTGAATTGCTGTACGCAACGCAAG TCAACCATGAGCGCAATATATCGGAGGGCTAAACCTAAA CCTAACC	MT-MT-WT Duplex Capture Probe Overhang
BLoli9192		GAGTCCACCACGATCGTGAATTGCTGTACGCAACGCAAG TCAACCATGAGCGCAATATATCGGAGGGCTAAACCTAAC CCTAAAC	MT-WT-MT Duplex Capture Probe Overhang
BLoli9193		GAGTCCACCACGATCGTGAATTGCTGTACGCAACGCAAG TCAACCATGAGCGCAATATATCGGAGGGCTAACCCTAAA CCTAAAC	WT-MT-MT Duplex Capture Probe Overhang
BLoli9194		GAGTCCACCACGATCGTGAATTGCTGTACGCAACGCAAG TCAACCATGAGCGCAATATATCGGAGGGCTAAACCTAAA CCTAAAC	MT-MT-MT Duplex Capture Probe Overhang
BLoli9202		CCCTCCGATATATTGCGCTCATGGTTGACTTGCGTTGCGT ACAGCAATTCACGATCGTGGTGGACTCTATGACCGTTCA/ 3BIO/	Duplex Capture Probe Biotinylated Reverse Complement

Supplemental Table 4. Illumina WGS and data processing statistics.

Patient	Relation to Index	Tissue	Trimmed Read Pairs	Number of Mapped Reads	Number of Unmapped Reads	Number of Reads Properly Paired	Number of Reads Duplicated	Average Insert Size	Average Read Quality
III.3	Index	Blood	234,094,487; (91.49%)	468116961; (99.98%)	72013; (0.015%)	457809170; (97.78%)	42869536; (9.16%)	253.8	33.1
II.3	Maternal Aunt	Blood	195,954,466; (87.56%)	391859903; (99.99%)	49029; (0.013%)	385933568; (98.48%)	44809314; (11.43%)	248.9	33.1
III.2	Twin Sister*	Blood	239,248,311; (85.25%)	478439806; (99.99%)	56816; (0.012%)	472921724; (98.83%)	72473881; (15.15%)	225.7	33.3
III.1	Older Sister	Blood	202,377,991; (84.28%)	404710348; (99.99%)	45634; (0.011%)	399835248; (98.78%)	60535146; (14.96%)	226.2	33.3
II.2	Mother	Blood	241,665,882; (84.70%)	483276195; (99.99%)	55569; (0.011%)	477565122; (98.81%)	80779837; (16.71%)	225.3	33.3
III.4	Maternal Cousin	Blood	317,011,662; (82.29%)	633928129; (99.98%)	95195; (0.015%)	625053888; (98.59%)	87667718; (13.83%)	245.1	33.1
Control	Control 0079	Blood	243,798,284; (84.56%)	487521462; (99.98%)	75106; (0.015%)	479477808; (98.33%)	57120290; (11.71%)	249.1	33.2
Control	Control 1262	Blood	186,679,556; (82.70)	373243431; (99.97%)	115681; (0.031%)	365931556; (98.01%)	42770837; (11.46%)	243	33.2
III.3	Index	Fibroblast	240,421,612; (79.03%)	480691543; (99.97%)	151681; (0.032%)	453753242; (94.37%)	75190045; (15.64%)	252.9	33.2
III.2	Twin Sister	Fibroblast	324,981,926; (70.54%)	649015086; (99.85%)	948766; (0.146%)	639143388; (98.34%)	86782105; (13.35%)	239.4	33.1
Control	Control 0079	Fibroblast	242,948,293; (78.73%)	485769276; (99.97%)	127310; (0.026%)	452681542; (93.16%)	74070765; (15.24%)	255	33.2
Control	Control 1262	Fibroblast	253,847,198; (77.09%)	507626168; (99.99%)	68228; (0.013%)	481386052; (94.82%)	76144737; (15%)	234	33.2

*Post bone marrow transplant - Data represents individual II.1; the father of the index Patient

Supplemental Table 5. Illumina ribo-depleted RNA sequencing and data processing statistics.

Patient	Relation to Index	Raw Read Pairs	Trimmed Read Pairs	Uniquely Mapping Reads	Multi-Mapping Reads	Unmapped Reads	Number of Reads Duplicated	Average Read Quality	Average Read Length
III.3	Index	195,119,453	195,029,025; (99.95%)	161,263,476; (82.69%)	23,365,086; (11.98%)	10,165,195; (5.22%)	116,107,839; (29.77%)	33.2	80
II.3	Aunt	243,935,299	243,894,905; (99.98%)	164,157,899; (67.31%)	69,030,806; (28.30%)	8,642,614; (3.55%)	170,895,810; (35.03%)	33.8	75
III.1	Older Sister	242,336,236	242,295,216; (99.98%)	123,655,860; (51.04%)	106,165,637; (43.82%)	9,353,127; (3.86%)	239,773,900; (49.48%)	33.7	75

Supplemental Table 6. Telomeric sequence related statistics from Illumina WGS.

		Illumina WGS								
**Patient**	**Tissue**	**Raw Read Count**	**Fully Telomeric Sequences**	**% Reads Containing**	**% Mutant Repeats**	**Mutant Consecutivity**	**Mutant Processivity**	**% WT Repeats**	**WT Consecutivity**	**WT Processivity**
III.3	Blood	511,740,350	11,012	50.63	9.51	0.676	0.352	86.53	0.734	0.467
III.3	Fibroblasts	608,410,276	12,144	44.84	8.48	0.606	0.211	87.7	0.721	0.441
III.2	Fibroblasts	921,377,370	10,999	38.45	7.15	0.598	0.196	88.61	0.725	0.449
III.1	Blood	480,265,836	12,054	27.62	4.73	N/A	N/A	92.17	N/A	N/A
II.2	Blood	570,609,452	18,394	41.89	8.27	0.666	0.333	88.58	0.724	0.447
III.2	Blood	561,334,728	12,788	10.75	0.94	N/A	N/A	96.27	N/A	N/A
II.3	Blood	447,610,790	14,122	43.32	8.5	0.702	0.404	88.22	0.733	0.466
III.4	Blood	770,479,046	34,962	29.7	5.04	N/A	N/A	91.9	N/A	N/A
II.4	Blood	N/A	N/A	N/A	N/A	N/A	N/A	N/A	N/A	N/A
Control	Blood	576,640,378	19,741	11.87	0.87	N/A	N/A	96.47	N/A	N/A
Control	Fibroblasts	617,150,848	15,946	8.17	0.62	N/A	N/A	96.98	N/A	N/A
Control	Blood	451,480,084	19,924	10.52	0.71	N/A	N/A	96.97	N/A	N/A
Control	Fibroblasts	658,595,704	38,551	6.31	0.46	N/A	N/A	97.67	N/A	N/A

Supplemental Table 7. Telomeric sequence related statistics from Nanopore Telomere Sequencing.

		Nanopore Telomere Enriched Sequencing						
**Patient**	**Tissue**	**Fully Telomeric Sequences**	**% Mutant Repeats**	**Mutant Consecutivity**	**Mutant Processivity**	**% WT Repeats**	**WT Consecutivity**	**WT Processivity**
II.3	Blood	6,266	4.67	0.699	0.380	82.81	0.961	0.923
III.4	Blood	3,007	2.62	N/A	N/A	88.45	N/A	N/A
II.4	Blood	5,464	0.38	N/A	N/A	90.59	N/A	N/A
