# Supplementary material for: RTEL1 mutation modifies dyskeratosis congenita caused by a telomerase RNA template mutation
Source: Nat Commun. 2026 Aug 26;17:9054. doi: 10.1038/s41467-026-76650-w (PMC13518968; doi:10.1038/s41467-026-76650-w)
Supplement: Supplementary file 2 [file 41467_2026_76650_MOESM2_ESM.pdf]

nature portfolio

Corresponding author(s): Dr. Peter Baumann and Dr. Susann Schweiger

Last updated by author(s): Jul 9, 2026

Reporting Summary

Nature Portfolio wishes to improve the reproducibility of the work that we publish. This form provides structure for consistency and transparency in reporting. For further information on Nature Portfolio policies, see our Editorial Policies and the Editorial Policy Checklist.

Statistics

For all statistical analyses, confirm that the following items are present in the figure legend, table legend, main text, or Methods section.

n/a	Confirmed	
☐	☒	The exact sample size (*n*) for each experimental group/condition, given as a discrete number and unit of measurement
☐	☒	A statement on whether measurements were taken from distinct samples or whether the same sample was measured repeatedly
☐	☒	The statistical test(s) used AND whether they are one- or two-sided<br>*Only common tests should be described solely by name; describe more complex techniques in the Methods section.*
☒	☐	A description of all covariates tested
☐	☒	A description of any assumptions or corrections, such as tests of normality and adjustment for multiple comparisons
☐	☒	A full description of the statistical parameters including central tendency (e.g. means) or other basic estimates (e.g. regression coefficient) AND variation (e.g. standard deviation) or associated estimates of uncertainty (e.g. confidence intervals)
☐	☒	For null hypothesis testing, the test statistic (e.g. *F*, *t*, *r*) with confidence intervals, effect sizes, degrees of freedom and *P* value noted<br>*Give P values as exact values whenever suitable.*
☒	☐	For Bayesian analysis, information on the choice of priors and Markov chain Monte Carlo settings
☒	☐	For hierarchical and complex designs, identification of the appropriate level for tests and full reporting of outcomes
☒	☐	Estimates of effect sizes (e.g. Cohen's *d*, Pearson's *r*), indicating how they were calculated

Our web collection on statistics for biologists contains articles on many of the points above.

Software and code

Policy information about availability of computer code

Data collection	QuantStudio Real-Time PCR Software v1.3 was used for the collection of qPCR related data
Data analysis	DNA sequencing eads were trimmed using Trimmomatic47,48 PE v0.39 with the following parameters: SLIDINGWINDOW:5:15, LEADING:10, TRAILING:10, MINLEN:100, and ILLUMINACLIP:TruSeq3-PE.fa:2:20:10. Alignment to human reference GRCh38 was performed using BWA MEM49 v0.7.17.<br>RNA sequencing reads were trimmed using Trimmomatic PE47 v0.39 with the following parameters SLIDINGWINDOW:5:10, LEADING:5, TRAILING:5, MINLEN:18, and ILLUMINACLIP:TruSeq3-PE.fa:2:20:10. Trimmed reads were aligned to human reference GRCh38 using STAR50 v2.7.10b and default parameters.<br>Nanopore derived telomeric sequences were basecalled with Dorado v0.7.0 and analyzed using TARPON v1.0.0 and custom code found at github.com/baumannlab<br><br>All gels were quantied using ImageJ software v1.52i.

For manuscripts utilizing custom algorithms or software that are central to the research but not yet described in published literature, software must be made available to editors and reviewers. We strongly encourage code deposition in a community repository (e.g. GitHub). See the Nature Portfolio guidelines for submitting code & software for further information.

Data

Policy information about availability of data

All manuscripts must include a data availability statement. This statement should provide the following information, where applicable:

- Accession codes, unique identifiers, or web links for publicly available datasets
- A description of any restrictions on data availability
- For clinical datasets or third party data, please ensure that the statement adheres to our policy

The raw sequencing data supporting the findings of this study have been deposited in the European Genome-phenome Archive (EGA), which is hosted by the EBI and CRG. The data supporting the findings of this study contain sensitive human participant information and cannot be made publicly available due to ethical and privacy restrictions. The data is available under restricted access via dataset accession EGAS50000001644[https://ega-archive.org/dacs/EGAC50000000925]. Data can be accessed for research purposes by authorized individuals. Further details on how to access the data, including the Data Access Committee (DAC) policy and Data Access Agreement requirements, can be found directly on the dataset page at https://ega-archive.org/". Source data are provided with this paper.

Research involving human participants, their data, or biological material

Policy information about studies with human participants or human data. See also policy information about sex, gender (identity/presentation), and sexual orientation and race, ethnicity and racism.

Reporting on sex and gender	No sex- or gender-based analysis was completed in this study nor was it taken into consideration during sample collection. This project analyzes a rare mutation only known to be present within this family and one additional family in the US and therefore relevant samples are limited in scope. Therefore, no large-scale sex or gender based analysis was completed.
Reporting on race, ethnicity, or other socially relevant groupings	No socially constructed categorization variables are used within this study.
Population characteristics	No relevant population level co-variate statistics were included in statistical analysis as no population level statistics were performed in this study.
Recruitment	Participants were recruited due to their relationship with the index patient (III.3). No self-inflicted bias is present within sample selection.
Ethics oversight	This project was overseen by the Clinical Ethics Committee of the Johannes Gutenberg University Medical Center

Note that full information on the approval of the study protocol must also be provided in the manuscript.

Field-specific reporting

Please select the one below that is the best fit for your research. If you are not sure, read the appropriate sections before making your selection.

☒ Life sciences ☐ Behavioural & social sciences ☐ Ecological, evolutionary & environmental sciences

For a reference copy of the document with all sections, see nature.com/documents/nr-reporting-summary-flat.pdf

Life sciences study design

All studies must disclose on these points even when the disclosure is negative.

Sample size	7 Patients were included in this study. No sample size calculations were performed as this is a rare-disease mutant found within only one family in this study and one family in ta previously published study. All accessible samples were analyzed. For in vitro experiments, when possible, triplicates were used for statistical purposes. When no statistical tests were required duplicates were performed to verify replicable results.
Data exclusions	No data was excluded from the analysis.
Replication	All non-sequencing based experiments were replicated in triplicate when used for statistical calculations. All attempts at replication were successful
Randomization	No randomization was performed as it is not applicable for this study.
Blinding	As this was not a large cohort study with different population groups, blinding was not performed. However, every sample was analyzed using the same methodology regardless of underlying patient genotype

Reporting for specific materials, systems and methods

We require information from authors about some types of materials, experimental systems and methods used in many studies. Here, indicate whether each material, system or method listed is relevant to your study. If you are not sure if a list item applies to your research, read the appropriate section before selecting a response.

Materials & experimental systems

n/a	Involved in the study	
☐	☒	Antibodies
☐	☒	Eukaryotic cell lines
☒	☐	Palaeontology and archaeology
☒	☐	Animals and other organisms
☐	☒	Clinical data
☒	☐	Dual use research of concern
☒	☐	Plants

Methods

n/a	Involved in the study	
☒	☐	ChIP-seq
☒	☐	Flow cytometry
☒	☐	MRI-based neuroimaging

Antibodies

Antibodies used	α-HA-Tag Mouse Diluted 1:10000 Invitrogen #261831MG Lot: YB370961<br>α-Tubulin Mouse Diluted 1:4000 Merck #T5168 Batch: 0000126956<br>α-Mouse-HRP Goat Diluted 1:10000 Invitrogen #31430 Lot: 3408712<br>CD45RA BV 421 / Clone: HI100 / Biolegend Cat Nr. 304130 / Lot# B359105 / 135µg/ml<br>CD20 eFluor 615/ Clone: L26 / eBioscience Cat. Nr. 42-0202-82 / Lot# 2567403 / 18µg/ml<br>CD57 PE / Clone: NK-1 / BD Cat. Nr. 560844 / Lot# 2245781 / 2,25µg/ml
Validation	Invitrogen # 31430 has been validated for use in Western blot, immunohistochemistry (IHC), ELISA, and immunoprecipitation (IP) applications.<br>Merck #T5158 was validated using Merck Enhanced Validation Techniques. Please see the supplier website for additional information. (https://www.sigmaaldrich.com/DE/en/technical-documents/technical-article/protein-biology/immunohistochemistry/antibody-enhanced-validation)<br>Invitrogen #26183 is specific for the HA peptide YPYDVPDYA. The antibody has been used successfully in Western blot and immunoprecipitation applications. This Antibody was verified by Relative expression to ensure that the antibody binds to the antigen stated.

Eukaryotic cell lines

Policy information about cell lines and Sex and Gender in Research

Cell line source(s)	HeLa Cells (ATCC-CCL-2.2)<br>WI-38 VA13 (ATCC-CCL-75.1)<br>Primary Fibroblasts (Male and Female)
Authentication	Commercially available cell lines were purchased directly from ATCC and therefore no authentication was needed. Patient derived fibroblast samples were authenticated based on the presence/absence of the C50>A hTR Mutation described within this paper.
Mycoplasma contamination	No cell lines were tested for mycoplasma contamination
Commonly misidentified lines (See ICLAC register)	No commonly misidentified cell lines are used within this study. Cell lines were purchased directly from ATCC.

Clinical data

Policy information about clinical studies

All manuscripts should comply with the ICMJE guidelines for publication of clinical research and a completed CONSORT checklist must be included with all submissions.

Clinical trial registration	This study has been reviewed and approved by the Clinical Ethics Committee (CEC) of the Johannes Gutenberg-University Medical Center. However, it is not a clinical trial and therefore no clinical trial registration was completed.
Study protocol	No protocol is required as determined by the CEC of the University Medical Center Mainz
Data collection	Data was collected during routine patient visits as standard diagnostic treatment protocols
Outcomes	N/A

Plants

Seed stocks	N/A
Novel plant genotypes	N/A
Authentication	N/A
