# Supplementary material for: RTEL1 mutation modifies dyskeratosis congenita caused by a telomerase RNA template mutation
Source: Nat Commun. 2026 Aug 26;17:9054. doi: 10.1038/s41467-026-76650-w (PMC13518968; doi:10.1038/s41467-026-76650-w)
Supplement: Supplementary file 3 [file 41467_2026_76650_MOESM3_ESM.pdf]

nature portfolio

Peer Review File

RTEL1 Mutation Modifies Dyskeratosis Congenita Caused By A Telomerase RNA Template Mutation

Corresponding Author: Professor Peter Baumann

This file contains all reviewer reports in order by version, followed by all author rebuttals in order by version.

Version 0:

Reviewer comments:

Reviewer #1

(Remarks to the Author)
In their manuscript titled "RTEL1 mutation as a modifier of dyskeratosis congenita in a family with a Telomerase RNA template mutation and variant telomeric repeats" the authors identify a patient with a severe telomere biology disorder that results from a mutation of telomerase RNA (TR) template region (C50>A), which alters the telomeric repeat sequence. Using biochemical assays, the authors demonstrate that telomerase containing TR with the C50>A mutation has reduced processivity, which was confirmed by sequencing of the telomeric DNA from patient samples using illumina and nanopore sequencing. Strikingly, mutant telomeric repeats are inherited by the offspring of carriers of the C50>A mutation and the mutant repeat content is simply "diluted" from ~8% to ~4%, which suggests limited overall turnover of telomeric DNA across generations. In addition to the C50>A mutation the authors also identify a nonsense mutation in RTEL1 in family members affected by severe telomere biology disorders suggesting a compounding effect of the two heterozygous mutations. The quality of the data is high and the findings of this work is highly impactful for our understanding of telomere biology and the etiology of telomere biology disorders. I support publication of this manuscript in its current form. The authors could expand their discussion of the telomerase processivity analysis. It appears that telomerase processivity is bi-phasic for the wildtype repeats, with a rapidly decaying initial phase and a more reduction in telomerase dissociation after that, which is what the linear regression in the figure represents.

(Remarks on code availability)

Reviewer #2

(Remarks to the Author)
This study extends characterization of a telomerase RNA (TERC) template mutation, r.C50>A, identified in a multi-generational family presenting with dyskeratosis congenita (DC) and idiopathic pulmonary fibrosis (IPF). The mutation causes substitution of the canonical telomeric repeat GGTTAG with the variant GTTTAG that was previously characterized in Hinchie Nature Communications 2024. The current manuscript using a combination of biochemistry, genetic analysis, and multiple forms of DNA sequencing to characterize this unique family. The current studies suggest that the template variant causes a decrease in processivity and confirms several of the previously reported phenotypes. One of the most remarkable findings of the current manuscript is the ONT long-read sequencing data that suggestes that mutant repeats are maintained at ~8% of total telomeric sequence across heterozygous carriers in two generations, and persist at ~4% in descendants who inherited only wild-type TERC. This implies remarkably slow turnover of telomeric sequence and places new constraints on models of telomere dynamics. In addition, the investigators report a co-segregating RTEL1 nonsense mutation (Q1178x) of paternal origin, likely subject to nonsense-mediated decay. Individuals carrying only the RTEL1 mutation presented with very short telomeres but no overt DC phenotype, whereas co-inheritance of both the TERC and RTEL1 mutations coincided with severe early-onset DC in two siblings. These findings underscore the importance of multigenic evaluation in telomere biology disorders and highlight how interactions between distinct telomere maintenance pathways can profoundly modulate disease penetrance. Overall, this is a well written and interesting manuscript that confirms the previous characterization of the C50>A template variant and adds novel insights that can only be obtained by studying these extraordinarily rare individuals.
There are several points that should be addressed in the manuscript that are listed below.
1. The authors should contextualize their findings throughout the results section to help the reader know when their findings

are confirmatory vs distinct from previous studies. Given that the variant identified by the authors was reported more than 18 months ago (Hinchie Nature Communications 2024), the authors should acknowledge where their findings are similar and where they differ.
2. Some of the authors findings appear to be contradictory. In figures 3D-E, the investigators report that the presence of a variant in the telomere DNA causes reduced engagement of wild type and variant telomerase. Yet when the investigators mix the two enzymes 1:1, there is no effect on telomerase processivity or total activity (figure 2b). Moreover, telomerase has been reported to extend non-telomeric sequences (Harrington Nature 1991). Can the authors address why a variant primer inhibits telomerase engagement, but when a variant sequence is added by telomerase, it does not influence engagement? In addition, why does a mutant primer reduce engagement of a mutant telomerase (line 247) given that the mutant telomerase would likely pair perfectly with this substrate?
3. The direct assays here do not include processivity factors that likely will influence telomerase activity in vivo (i.e. POT1/TPP1). The presence of a variant sequence at the terminal end of substrate DNA has been reported to increase telomerase activity by preventing the binding of POT1 (Wang Nature 2007, The POT1–TPP1 telomere complex is a telomerase processivity factor). Can the authors test the influence of these factors in their assays?
4. The observation that 43% of all junctions between WT-Mut sequence contain an extra G is striking. The authors speculate that this is due to slippage during DNA replication and is found more frequently at internal sequences. Can the authors investigate internal variant sequences to test if this phenomenon is more common in the oldest (most internal) variant repeats? Furthermore, variant sequences found in internal telomeric repeats or this sequence more generally should be found throughout the genome. An alternative explanation is the alignment of a variant sequence with a G-strand telomere that terminates with the 5`-GGTTAGGGTT-3` sequence. Sfeir Molecular Cell 2005 reported that about 25% of telomeres terminate with the 5`-GGTTAGGGTT-3` sequence. Given that this sequence would align almost perfectly with the variant telomerase (one G-U pair in DNA/RNA hybrid) and generate the reported sequence, it seems plausible that this could contribute to the frequently observed GGTTAG–G–GTTTA sequence.
5. The authors state that the presence of a variant sequence did not affect the binding of shelterin (line 542-544), but do not show any binding data. The reference cited by the authors demonstrated that the variant sequence did inhibit POT1 binding. Previous studies have shown that TRF1 binds with high specificity to the telomeric sequence, but can tolerate long tracts of non-telomeric sequences between two canonical sequences (Bianchi EMBO 1999).

(Remarks on code availability)

Reviewer #3

(Remarks to the Author)
Deimler, Orioli, et al present a clinical and molecular study of the impact of a heterozygous template mutation C50>A in the telomerase RNA component gene TERC. The pedigree studied also is found to carry a likely pathogenic het. truncating mutation in the RTEL1 gene p.Q1778X. From a clinical standpoint, co-inheritance of these mutations is implicated in a severe phenotype of dyskeratosis congenita in the proband and his twin, with less prominent or later onset manifestations of telomere diseases in family members carrying either het. mutation alone. Detailed molecular studies are performed including telomere length, telomere content, and impacts of the C50>A template mutation on telomerase, both in vitro and by analysis of telomeric sequences from patient samples via Illumina and Nanopore sequencing technologies.
Important findings that are well-described include:
(1) oligogenic contributions to TBDs that are implicated in disease manifestations and categorization
(2) incorporation and tolerance of variant telomere sequences due to the TERC mutation, in blood and fibroblast DNA, but not uniformly manifesting in very short telomeres on diagnostic testing
(3) inheritance of variant telomere repeat sequences and their relative stability across generations, irrespective of TERC mutation co-inheritance
(4) enriched distal location of variant repeats along the telomere, if the TERC mutation is co-inherited
(5) decreased activity of template mutant telomerase both in vitro in direct telomerase activity assays and in vivo based on consecutive variant repeat sequences in genomic DNA

Other findings of potential importance but less well-described/-explained include:
(1) potentially successful long-term hematopoietic reconstitution using a donor carrying a het. RTEL1 mutation
(2) detection of variant telomere repeat sequences with extra or missing G residues in patient genomic DNA, in the setting of WT plus C50>A mutant telomerase
(3) increased variant repeat content in longer telomeres

Overall, major strengths of the manuscript include (1) breadth of nuanced clinical to molecular analyses by a team of experts across fields, and (2) insights gained from technologies applied to patient samples that open new opportunities. Weaknesses include: (1) lack of novelty of some of these observations; (2) data interpretation in certain experiments including long-read sequencing due to technical concerns; (3) a narrative that is difficult to follow as it switches between clinical and molecular descriptions.

Major critiques:
1) Novelty of TERC C50>A and effects on telomerase in vitro and in cells.
a. Hinchie et al, 2024 also describe the TERC C50>A variant in a family with telomere biology disorder, and similarly describe reduced processivity, the potential shifted nucleotide addition pause site (NAPS), and association of variant repeats with longer telomeres due to inhibition of POT1/shelterin binding. This paper should be cited in the appropriate sections of the results/discussion when similar findings/ concepts are presented. Currently it is briefly mentioned in the

discussion, lines 525-537
b. “Processivity” should be strictly defined as degree of extension after a single binding event. In several parts of the manuscript both in vitro and in DNA from cells, what is being called processivity may be indistinguishable from subsequent extension of a released product. This should be clarified, perhaps referring to data from sequencing as “apparent processivity”.
c. As the authors point out, “processivity” analysis of WT telomerase (Fig. 2g) based on Illumina sequencing is significantly hampered by the 150 bp read length. For clarity, the Illumina analysis could be omitted, given that a more robust estimation is subsequently provided by ONT.

2) Long-read sequencing. The most novel and interesting analyses are the Nanopore studies of II.3, II.4, III.4 including localization of variant repeat along the telomere. There are several opportunities for new insights here, but some technical concerns.
a. The mechanism of “extra” and “missing” G residues is interesting but unexplained. Based on the relative distribution along the telomere in Nanopore sequencing the authors conclude this is due to DNA replication. Does the Nanopore capture method account for the “extra” and “missing” G sequences?
b. Is the very last repeat captured? What proportion of those are variants? What is the last base? If there is a change in the nucleotide addition pause site, and less engagement and processivity of the variant repeat, one would expect that the terminal repeat would frequently end GGTTTA, instead of GGTTAG based on prior studies (e.g. Sfeir et al, 2005; Hinchie et al, 2024). If this is not profiled in the current sequencing, it should be shown by another method.
c. It would be interesting to assign and understand chromosome arm level variant repeat distributions, which should be available in the existing data. Is there a correlation of variants with chromosome arm, independent of length?
d. Even more interesting would be to see variant content based on chromosome inheritance. In III.4, is there evidence of cross over from the maternally-inherited chromosomes?
e. How does one interpret the detection of variants above background at the end of the telomere in III.4 in Fig. 6d, which implies these variants have not undergone any turnover?
f. Also in Fig.6d, the analysis slides only to 4 kb from capture probe. While this distribution is reasonably representative for II.3, it is inadequate for III.4 (based on Fig 6a). It would be worthwhile to extend this into the subtelomere, perhaps scaled as percentage along the telomere instead of by KB.

3) Clinical narrative and RTEL1 finding.
a. Lines 309-358 – while the narrative of how the RTEL1 mutation was found could be instructive to clinicians, it is confusing. Especially the subsequent genotyping of III.2, when her blood sample has subsequently been used to provide II.1’s genotypes in Fig. 5c (in conflict with the statement in lines 322-323). I suggest re-writing the clinical narrative, ideally contained in one section of the paper to provide the manifestations, genotypes, and clinical course more succinctly.
b. Except for potentially explaining the severity of clinical manifestations in III.2 and III.3, the RTEL1 mutation finding does not impact the molecular studies on the TERC template and telomere variants, which seem to be the major focus of the manuscript. There could be an argument for describing this finding and potential genotype/phenotype correlations (which are interesting but also variable), transplant courses, etc, in a separate manuscript.
c. Oligogenic contributions to TBD manifestations have been previously reported and should be discussed/cited (e.g. PMID 26024875).

Minor comments:
1. Line 105, underlined letters in RTEL1 are incorrect
2. Lines 311 and 387, should refer to wild-type TERC, not WT telomerase
3. Line 319, “Clinvar one star” needs more explanation
4. Lines 506-526 re template variant studies: it may be worthwhile including reference/discussion of Jongmans et al (PMID 22341970), showing recurrent somatic genetic reversion in the setting of template domain mutations (del.n.54-57).
5. Lines 559-566, do not contribute meaningfully in this manuscript

(Remarks on code availability)

Reviewer #4

(Remarks to the Author)
I co-reviewed this manuscript with one of the reviewers who provided the listed reports. This is part of the Nature Communications initiative to facilitate training in peer review and to provide appropriate recognition for Early Career Researchers who co-review manuscripts.

(Remarks on code availability)

Version 1:

Reviewer comments:

Reviewer #1

(Remarks to the Author)

The reviewers have addressed the concerns raised by reviewers 2 and 3 and I support publication of this manuscript.

(Remarks on code availability)

Reviewer #2

(Remarks to the Author)
The authors have made extensive modifications to their manuscript and provided an thoughtful response to my concerns. I support the support the publication of this manuscript.

(Remarks on code availability)

Reviewer #3

(Remarks to the Author)
Deimler, Orioli, and colleagues present a substantially revised manuscript highly responsive to our critiques. The clinical narrative and insights therein are easier to digest and now positioned to amplify the rigorous molecular analyses that follow. It is appreciated that technical elements such as retaining a comparison of Illumina and Nanopore sequencing and identifying artifacts from 3’ fill-in during telomere end capture are helpful as the field adopts long-read telomere sequencing.

The additional analysis of III.4 (who has inherited variant telomere repeats but not the TERC mutation) identifying increased G insertion events between two mutant repeats compared to II.3 is an interesting observation and strongly supports a DNA replication “slippage” rather than mutant telomerase mechanism.

As one point of clarification for the new Fig. 6j and text lines 470-479 regarding the observation of decreased mutant repeat tract length: we infer that this is because of the increased DNA replication slippage events between mutant repeats, i.e. as these are prone to harbor extra Gs over time, they do not get “counted” in a consecutive repeat tract. Thus there is a technical underestimation of apparent mutant repeat processivity in an individual like this, because the algorithm detects fewer pure consecutive mutant repeats. Is that correct?

If so, it may be worthwhile clarifying the sentence in lines 475-477 which seems to indicate that there is actually reduced processivity. Then also, in line 477-479, perhaps this should state “another unexpected mechanism by which mutant telomere sequences contribute to accelerated telomere dysfunction” rather than “shortening”, if they are indeed not shorter but simply composed of more heterogeneous telomeric sequences (extra Gs) over time. Finally, as a way to further clarify/strengthen this observation, the authors might consider performing an additional analysis where they modify their mutant tract length calling pipeline to tolerate G insertions. Based on their hypothesis that DNA replication leads to G insertions, it would be expected that altering the analysis in this way would eliminate the mutant tract length discrepancy between II.3 and III.4.

Minor comment:
-line 142 – should be RTEL1 in italics

(Remarks on code availability)

Reviewer #4

(Remarks to the Author)
I co-reviewed this manuscript with one of the reviewers who provided the listed reports. This is part of the Nature Communications initiative to facilitate training in peer review and to provide appropriate recognition for Early Career Researchers who co-review manuscripts.

(Remarks on code availability)

Open Access This Peer Review File is licensed under a Creative Commons Attribution 4.0 International License, which permits use, sharing, adaptation, distribution and reproduction in any medium or format, as long as you give appropriate credit to the original author(s) and the source, provide a link to the Creative Commons license, and indicate if changes were made.
In cases where reviewers are anonymous, credit should be given to 'Anonymous Referee' and the source.
The images or other third party material in this Peer Review File are included in the article's Creative Commons license, unless indicated otherwise in a credit line to the material. If material is not included in the article's Creative Commons license and your intended use is not permitted by statutory regulation or exceeds the permitted use, you will need to obtain permission directly from the copyright holder.
To view a copy of this license, visit https://creativecommons.org/licenses/by/4.0/

REVIEWER COMMENTS

Reviewer #1 (Remarks to the Author):

In their manuscript titled "RTEL1 mutation as a modifier of dyskeratosis congenita in a family with a Telomerase RNA template mutation and variant telomeric repeats" the authors identify a patient with a severe telomere biology disorder that results from a mutation of telomerase RNA (TR) template region (C50>A), which alters the telomeric repeat sequence. Using biochemical assays, the authors demonstrate that telomerase containing TR with the C50>A mutation has reduced processivity, which was confirmed by sequencing of the telomeric DNA from patient samples using illumina and nanopore sequencing. Strikingly, mutant telomeric repeats are inherited by the offspring of carriers of the C50>A mutation and the mutant repeat content is simply "diluted" from ~8% to ~4%, which suggests limited overall turnover of telomeric DNA across generations. In addition to the C50>A mutation the authors also identify a nonsense mutation in RTEL1 in family members affected by severe telomere biology disorders suggesting a compounding effect of the two heterozygous mutations. The quality of the data is high and the findings of this work is highly impactful for our understanding of telomere biology and the etiology of telomere biology disorders. I support publication of this manuscript in its current form.

We sincerely thank the reviewer for their thoughtful and thorough evaluation of our manuscript. We appreciate their comments on the high quality of the data and referring to this work as "highly impactful for our understanding of telomere biology and the etiology of telomere biology disorders". We appreciate this reviewer's support for publication of "this manuscript in its current form".

The authors could expand their discussion of the telomerase processivity analysis. It appears that telomerase processivity is bi-phasic for the wildtype repeats, with a rapidly decaying initial phase and a more reduction in telomerase dissociation after that, which is what the linear regression in the figure represents.

We have expanded the result section, lines [401-405], to note that WT processivity exhibits a bi-phasic behavior with distinct slopes detected between 0 and ~75 repeats and 75+ repeats. ["Additionally, long read sequencing suggests that wild-type telomerase may exhibit bi- or tri-phasic processivity behavior with a steeper slope at shorter repeat stretches that lessens as consecutive repeat number increases (*Fig.* 4h)."]

Reviewer #2 (Remarks to the Author):

This study extends characterization of a telomerase RNA (TERC) template mutation, r.C50>A, identified in a multi-generational family presenting with dyskeratosis congenita (DC) and idiopathic pulmonary fibrosis (IPF). The mutation causes substitution of the canonical telomeric repeat GGTTAG with the variant GTTTAG that was previously characterized in Hinchie Nature Communications 2024. The current manuscript using a combination of biochemistry, genetic analysis, and multiple forms of DNA sequencing to characterize this unique family. The current studies suggest that the template variant causes a decrease in processivity and confirms several of the

previously reported phenotypes. One of the most remarkable findings of the current manuscript is the ONT long-read sequencing data that suggestes that mutant repeats are maintained at ~8% of total telomeric sequence across heterozygous carriers in two generations, and persist at ~4% in descendants who inherited only wild-type TERC. This implies remarkably slow turnover of telomeric sequence and places new constraints on models of telomere dynamics. In addition, the investigators report a co-segregating RTEL1 nonsense mutation (Q1178x) of paternal origin, likely subject to nonsense-mediated decay. Individuals carrying only the RTEL1 mutation presented with very short telomeres but no overt DC phenotype, whereas co-inheritance of both the TERC and RTEL1 mutations coincided with severe early-onset DC in two siblings. These findings underscore the importance of multigenic evaluation in telomere biology disorders and highlight how interactions between distinct telomere maintenance pathways can profoundly modulate disease penetrance. Overall, this is a well written and interesting manuscript that confirms the previous characterization of the C50>A template variant and adds novel insights that can only be obtained by studying these extraordinarily rare individuals.

We thank the reviewer for their thoughtful and detailed evaluation of our manuscript and for highlighting the significance of our findings, in particular the evidence pointing at remarkably slow turnover of telomeric sequence and its implications for models of telomere dynamics. We concur with the reviewer regarding the importance of multigenic evaluation in telomere biology disorders and we are pleased that the reviewer finds our study well written and interesting.

There are several points that should be addressed in the manuscript that are listed below.

1. The authors should contextualize their findings throughout the results section to help the reader know when their findings are confirmatory vs distinct from previous studies. Given that the variant identified by the authors was reported more than 18 months ago (Hinchie Nature Communications 2024), the authors should acknowledge where their findings are similar and where they differ.

We have now more clearly contextualized our findings relative to the previously published study by Hinchie et al. This paper is now referenced earlier in the introduction [lines 96-105] and we have revised the Results section to clearly describe where our observations confirm previous findings and where we provide novel insights.

2. Some of the authors findings appear to be contradictory. In figures 3D-E, the investigators report that the presence of a variant in the telomere DNA causes reduced engagement of wild type and variant telomerase. Yet when the investigators mix the two enzymes 1:1, there is no effect on telomerase processivity or total activity (figure 2b).

The in vitro telomerase assays were performed under conditions of excess unlabeled primer (700 nM). Under these conditions, the majority of primer remains unextended throughout the incubation period, and we are largely observing single turnover events as each telomerase molecule engages a supplied wildtype telomeric primer rather than the far less abundant (mutant) telomerase extension products. In the

experiment in Fig. 2b (now Fig. 3b), where both mutant and wildtype telomerase RNA are present, the detrimental effect of terminal mutant repeats on the engagement step (primer binding) is therefore not apparent as only wildtype primer is provided, and mutant extension products are far less abundant than wildtype primer. In contrast, figure 5c,e examines the extension of wildtype versus mutant telomeric primers by wildtype telomerase. Here the reduced initial engagement of the mutant primer is apparent. Once this primer has been extended by wildtype telomerase, processivity is indistinguishable from that on wildtype primer (parallel slopes in 5e) consistent with realignment involving newly synthesized wildtype sequence regardless of the sequence of the starting primer. This has now been clarified in the text [lines 427-440] and figure labels.

Moreover, telomerase has been reported to extend non-telomeric sequences (Harrington Nature 1991). Can the authors address why a variant primer inhibits telomerase engagement, but when a variant sequence is added by telomerase, it does not influence engagement?

The reduced engagement of the C50>A variant primer compared to the wildtype sequence is explained by the mismatch between the thymidine at the -4 position of the primer and C56 in the alignment region of hTR. This weakens the initial primer – RNA interaction. In addition, the mutant primer ends in dA instead of dG, shortening the alignment by one nucleotide. These sequence permutations were chosen as they represent the most abundant dissociation products of mutant and wildtype telomerase, respectively.

```
GGTTAGGTTTA                 GGTTAGGGTTAG
      -|||                        |||||
      CAATCCCAATC                 CAATCCCAATC
```

When a variant sequence has been added by mutant telomerase, it does negatively affect subsequent engagement by the wildtype enzyme. The effect on wildtype telomerase is shown in original figure Fig. 3d/e (now Fig. 5b/c/d/e). The effect on mutant telomerase can be inferred from three observations: (i) the alignment region of C50A is identical to wildtype, (ii) a C50A/C56A double mutant described by Hinchie et al. has increased processivity, and (iii) low processivity of C50A is consistent with weaker interactions favoring dissociation over translocation. This schematic has now been added as Figure 5d.

In addition, why does a mutant primer reduce engagement of a mutant telomerase (line 247) given that the mutant telomerase would likely pair perfectly with this substrate?

A mutant telomerase as in C50A would – just like wildtype - show reduced levels of engagement to a mutant telomeric primer. The reason is illustrated below. The mutation does not impact the alignment region. Hinchie et al generated a double mutant (C50A/C56A) to compensate for the mismatch between a terminal mutant repeat in the primer and the alignment region. As expected, this second mutation "rescues" the engagement and processivity defect of the C50A mutation (Hinchie et al, Figure 2)

Wildtype telomerase engages mutant primer

```
GTTTAGGTTTA
       -|||
       CAATCCCAATC
```

C50A telomerase engages mutant primer

```
GTTTAGGTTTA
       -|||
       CAATCCAAATC
```

Double mutant generated by Hinchie et al. rescues alignment interaction:

```
GTTTAGGTTTA
       ||||
       AAATCCAAATC
```

To make this more transparent, we have edited the text as follows on lines 430-434; "To assess whether terminal variant repeats impaired engagement by wild-type telomerase, in vitro telomerase assays were conducted using wild-type $(GGTTAG)_3$ and mutant $(GGTTTA)_3$ primers. The variant primer consistently yielded fewer extension products than the wild-type primer (Fig. 5c, compare lanes 1-5 with lanes 6-10), consistent with a shorter annealing region as illustrated in Fig. 5d"

3. The direct assays here do not include processivity factors that likely will influence telomerase activity in vivo (i.e. POT1/TPP1). The presence of a variant sequence at the terminal end of substrate DNA has been reported to increase telomerase activity by preventing the binding of POT1 (Wang Nature 2007, The POT1–TPP1 telomere complex is a telomerase processivity factor). Can the authors test the influence of these factors in their assays?

The effects of the POT1-TPP1 complex as a known processivity factor for telomerase were already tested by Hinchie et al. The addition of POT1-TPP1 resulted in only a slight increase in repeat addition processivity for C50A, far smaller than for the wildtype enzyme. While some overlap in the results presented here and in Hinchie et al. is unavoidable, we have tried to focus this manuscript on expanding on previously published findings, rather than on confirming each result previously published. Following the reviewers' suggestion we have substantially revised the text to clearly lay out what was already published by the Alder group, what we confirm, and where our results differ and/or expand on prior findings.

4. The observation that 43% of all junctions between WT-Mut sequence contain an extra G is striking. The authors speculate that this is due to slippage during DNA replication and is found more frequently at internal sequences. Can the authors investigate internal variant sequences to test if this phenomenon is more common in the oldest (most internal) variant repeats?

We appreciate the reviewer's interest in this unexpected finding and their comment has prompted us to look more deeply into the positional information for the extra guanosines. The results are now presented in figure 6. In brief, examination of the

long read sequencing data revealed that the frequency of guanosine insertions between WT-MT and MT-MT decrease near the distal ends of telomeres, indicating that they indeed result from slippage during conservative DNA replication. We have further expanded this analysis to include data from individual III.4. Here, an increase in MT-MT G insertions and a decrease in the mutant telomeric repeat tract length further support that DNA replication is responsible for these changes (Fig 6f to i).

Furthermore, variant sequences found in internal telomeric repeats or this sequence more generally should be found throughout the genome. An alternative explanation is the alignment of a variant sequence with a G-strand telomere that terminates with the 5`-GGTTAGGGTT-3` sequence. Sfeir Molecular Cell 2005 reported that about 25% of telomeres terminate with the 5`-GGTTAGGGTT-3` sequence. Given that this sequence would align almost perfectly with the variant telomerase (one G-U pair in DNA/RNA hybrid) and generate the reported sequence, it seems plausible that this could contribute to the frequently observed GGTTAG–G–GTTTA sequence.

Alignment of a telomere ending in GGTTAGGGTT-3' in one of the three possible registers would indeed produce the observed G insertion between WT and MT repeats. Reference to this alternative explanation has now been included in the text [lines 459-460; "While we cannot exclude that the additional guanosine residue between WT and MT repeats may result from an alternative alignment of hTR (Fig. 6h)"]. However, the observation that G insertions are more prevalent at internal sites argues against a major contribution of this mechanism.

```
GGTTAGGGTTTAG        GGTTAGGGTTAG
   |-:|||||||         ||||||-||
  CAAUCCAAAUC         CAAUCCAAAUC
```

5. The authors state that the presence of a variant sequence did not affect the binding of shelterin (line 542-544), but do not show any binding data. The reference cited by the authors demonstrated that the variant sequence did inhibit POT1 binding. Previous studies have shown that TRF1 binds with high specificity to the telomeric sequence, but can tolerate long tracts of non-telomeric sequences between two canonical sequences (Bianchi EMBO 1999).

We did not perform binding assays with shelterin components as the results by Hinchie et al. already showed that the presence of mutant telomeric repeats did not cause an increase in TIF formation, unless an additional mutation was introduced at position 56 to processively add mutant repeats. This suggests that TRF1/2 either bind the mutant telomeric repeats directly, albeit with reduced affinity or TRF1/2 can span short mutant telomeric repeat tracts and bind the flanking wild-type telomeric repeats as shown in Bianchi et al. This has been expanded upon within the discussion [lines 565-577; "While the template mutation results in decreased telomerase engagement and processivity , the mutant telomeric repeat has also been shown to reduce the binding affinity of POT1[19]. This reduced POT1 binding may increase telomerase accessibility at mutant-containing telomeres, contributing to the long, mutant-rich telomeres reported here. Despite this effect on POT1 and the high sequence specificity of TRF1 and TRF2 for canonical telomeric repeats[47], the presence of the mutant sequences did not lead to an

increased DNA damage response in patient-derived cells[19]. This suggests that the shelterin complex retains sufficient protective function at GTTTAG-containing telomeres. The relatively low processivity of C50>A telomerase is likely to limit disruption of TRF1 and TRF2 binding, as the homodimers can bridge mutant tracts and interact with flanking wild-type telomeric repeats, as demonstrated in vitro for TRF1 binding two AGGGTT sites separated by non-telomeric DNA[47]. A detrimental effect of the mutant repeats on shelterin binding might be further mitigated by the guanosine insertions described here as they generate additional higher affinity binding sites for TRF1/2."]

Reviewer #3 (Remarks to the Author):

Deimler, Orioli, et al present a clinical and molecular study of the impact of a heterozygous template mutation C50>A in the telomerase RNA component gene TERC. The pedigree studied also is found to carry a likely pathogenic het. truncating mutation in the RTEL1 gene p.Q1778X. From a clinical standpoint, co-inheritance of these mutations is implicated in a severe phenotype of dyskeratosis congenita in the proband and his twin, with less prominent or later onset manifestations of telomere diseases in family members carrying either het. mutation alone. Detailed molecular studies are performed including telomere length, telomere content, and impacts of the C50>A template mutation on telomerase, both in vitro and by analysis of telomeric sequences from patient samples via Illumina and Nanopore sequencing technologies.
Important findings that are well-described include:
(1) oligogenic contributions to TBDs that are implicated in disease manifestations and categorization
(2) incorporation and tolerance of variant telomere sequences due to the TERC mutation, in blood and fibroblast DNA, but not uniformly manifesting in very short telomeres on diagnostic testing
(3) inheritance of variant telomere repeat sequences and their relative stability across generations, irrespective of TERC mutation co-inheritance
(4) enriched distal location of variant repeats along the telomere, if the TERC mutation is co-inherited
(5) decreased activity of template mutant telomerase both in vitro in direct telomerase activity assays and in vivo based on consecutive variant repeat sequences in genomic DNA

We thank the reviewer for their careful evaluation of our work and listing its important findings as well described.

Other findings of potential importance but less well-described/-explained include:
(1) potentially successful long-term hematopoietic reconstitution using a donor carrying a het. RTEL1 mutation
(2) detection of variant telomere repeat sequences with extra or missing G residues in patient genomic DNA, in the setting of WT plus C50>A mutant telomerase
(3) increased variant repeat content in longer telomeres

Overall, major strengths of the manuscript include (1) breadth of nuanced clinical to

molecular analyses by a team of experts across fields, and (2) insights gained from technologies applied to patient samples that open new opportunities. Weaknesses include: (1) lack of novelty of some of these observations; (2) data interpretation in certain experiments including long-read sequencing due to technical concerns; (3) a narrative that is difficult to follow as it switches between clinical and molecular descriptions.

We thank the reviewer for the detailed and constructive assessment of our manuscript. We appreciate the recognition of the manuscript's major strengths, including the breadth of clinical-to-molecular analyses performed by a multidisciplinary team and the insights gained from applying high-resolution sequencing technologies to patient samples. We acknowledge the reviewer's concerns regarding novelty, data interpretation, and narrative flow and have therefore performed major revisions and restructuring of the manuscript to (i) highlight where this study differs and expands on findings by Hinchie et al, (ii) address the technical concerns by expanding the long read sequencing data analysis and (iii) collapse all clinically relevant information into one section to improve narrative flow. We agree with the reviewer that "potentially successful long-term hematopoietic reconstitution using a donor carrying a het. RTEL1 mutation" is an interesting finding not expanded on in detail in this study. A separate case report, led by the clinicians who cared for patient III.2, will describe this aspect in more detail.

Major critiques:

1) Novelty of TERC C50>A and effects on telomerase in vitro and in cells.

a. Hinchie et al, 2024 also describe the TERC C50>A variant in a family with telomere biology disorder, and similarly describe reduced processivity, the potential shifted nucleotide addition pause site (NAPS), and association of variant repeats with longer telomeres due to inhibition of POT1/shelterin binding. This paper should be cited in the appropriate sections of the results/discussion when similar findings/ concepts are presented. Currently it is briefly mentioned in the discussion, lines 525-537

The Hinchie et al. paper is now referenced in the introduction [lines 93-105] and at several points throughout the results section and discussion. We have contextualized our findings and clearly state where results confirm prior work and where this study provides novel insights, including a major expansion of our long-read sequencing data analysis.

b. "Processivity" should be strictly defined as degree of extension after a single binding event. In several parts of the manuscript both in vitro and in DNA from cells, what is being called processivity may be indistinguishable from subsequent extension of a released product. This should be clarified, perhaps referring to data from sequencing as "apparent processivity".

We thank the reviewer for making this important distinction. The manuscript has been updated to clearly distinguish between telomerase processivity calculations from in vitro data and calculations based on the number of consecutive mutant telomeric repeats in sequencing data from patient DNA. In the calculations based on sequencing

data we distinguish between processivity (repeats added by the same enzyme molecule) and consecutivity (repeats located next to each other) analogous to Hinchie et al. [lines 367-373. "However, apparent processivity of mutant telomerase was lower within fibroblast derived samples for both III.3 (0.21) and III.2 (0.20) (*Fig*. 4c/d), an observation that hints at tissue specific differences in telomerase activity or telomere dynamics. The apparent processivity for wild-type telomerase was only modestly higher than for the mutant enzyme (*Fig*. 4e/f), but this analysis underestimated wild-type enzyme processivity due to the 150-nucleotide Illumina read-length limitation, which imposed an artificial upper boundary on the available repeat tract length data."]

c. As the authors point out, "processivity" analysis of WT telomerase (Fig. 2g) based on Illumina sequencing is significantly hampered by the 150 bp read length. For clarity, the Illumina analysis could be omitted, given that a more robust estimation is subsequently provided by ONT.

We would like to retain the Illumina analysis in the manuscript despite its shortcomings to (a) highlight the consistency between Illumina and ONT data in other respects (e.g. Fig 2d) and (b) illustrate the increasing value of long read sequencing for telomere research. Until recently, ONT has been hampered by low accuracy and the direct comparison between Illumina and ONT data provided here illustrates recent advances in flow cell chemistry and base calling algorithms. As telomere research increasingly embraces ONT sequencing, we believe that a few comparisons with established or complementary technology are valuable. Nevertheless, in response to the reviewer's comment, we have shortened the description of the Illumina data in the manuscript.

2) Long-read sequencing. The most novel and interesting analyses are the Nanopore studies of II.3, II.4, III.4 including localization of variant repeat along the telomere. There are several opportunities for new insights here, but some technical concerns.

a. The mechanism of "extra" and "missing" G residues is interesting but unexplained. Based on the relative distribution along the telomere in Nanopore sequencing the authors conclude this is due to DNA replication. Does the Nanopore capture method account for the "extra" and "missing" G sequences?

Nanopore telomere sequencing was performed using a 3-repeat capture probe composed of all possible permutations of WT and MT telomeric repeats. As mismatches between G-strand and capture probe may not prevent capture probe ligation, the 5' terminal three repeats are automatically removed from the analysis by TARPON (Deimler et al., 2026). The same issue does not arise during G-strand sequencing as the native DNA strand is sequenced. As the reviewer referred to the nanopore analysis of patient DNA as "most novel and interesting", we have expanded on this analysis in Fig. 6 to illustrate both the frequency and location of these events within the telomere. Guanosine insertions are less common near the distal end (where telomerase is active) than internally, indicating that they may arise during DNA replication instead of extension by telomerase. To further demonstrate this, we have analyzed telomere sequencing data from individual III.4, who has inherited mutant

telomeric repeats, but not C50>A TERC. Here, all mutant telomeric repeats have undergone multiple rounds of replication and none have been synthesized de novo by telomerase. Strikingly, the frequency of insertions between mutant telomeric repeats nearly doubles in comparison to her mother, who harbors the C50>A mutation. Furthermore, the length of mutant telomeric repeat stretches decreases over time, suggesting that DNA replication slippage results in contraction of mutant repeat tracts. These findings have now been included in the manuscript in Figure 6.

b. Is the very last repeat captured? What proportion of those are variants? What is the last base? If there is a change in the nucleotide addition pause site, and less engagement and processivity of the variant repeat, one would expect that the terminal repeat would frequently end GGTTTA, instead of GGTTAG based on prior studies (e.g. Sfeir et al, 2005; Hinchie et al, 2024). If this is not profiled in the current sequencing, it should be shown by another method.

The Hinchie et al. study nicely displays the telomere end sequences in Fig. 5f/g. They identify that the most frequent variant end is indeed GTTTA-3’ consistent with the one nucleotide shift of the NAPS. This is now referenced in the discussion [lines 540-543] “Additionally, when a telomere was identified to end in a mutant telomeric repeat, GTTTA was the most commonly identified end[19], consistent with in vitro DTAA findings reported here.” In the telomere enrichment protocol for nanopore sequencing, only the most common nucleotide permutation ending in GGTTAG and GTTTAG were used. The data is therefore unsuitable for the analysis of naturally occurring 3’ end distribution.

When performing additional controls for telomere enrichment and nanopore sequencing, we realized that the method does not reliably distinguish between bona fide terminal repeats and the product of fill-in synthesis during library preparation. The latter is a consequence of capture probe annealing with a gap between telomeric 3’ end and 5’ end of the capture probe (see figure below). During end-preparation and nick-repair, this small gap is filled in, thereby creating an artifactual 3’ end. This is evidenced by C50>A mutant telomeric repeats appearing as the most distal repeat (shown in red below) in some telomeres from wild type samples. The only reason this phenomenon of capture probe annealing in multiple registers is apparent here, is the use of wildtype/mutant telomeric repeat capture probes. It remains unclear how frequently this is happening when only permutations of the wild type telomeric repeat is used. Importantly, this phenomenon casts doubt on 3’ end sequence distributions derived from such telomere capture. As this potential artifact of the telomere end capture methodology may impact other studies as well, we have included the observation in the context of figure 2f, lines 316-326.

```
GGTTAGGGTTAGGGTTAGGGTTAGGGTTAGGGTTAGGTTTAGNNNNNNNNNNNN
                        ||||||||||||
                        CCAAUCCCAAUCCAAAUCNNNNNNNNNNNN
```

The Hinchie study uses C-tailing prior to telomere amplification and is thus not prone to the same artifact.

c. It would be interesting to assign and understand chromosome arm level variant repeat distributions, which should be available in the existing data. Is there a correlation of variants with chromosome arm, independent of length?

We agree that chromosome arm level variant repeat distributions would be interesting to examine; however, it has become clear that a sample-specific subtelomeric reference is essential for assigning chromosome arm identity to telomeric reads. Several recent studies have attempted to align telomeric ONT reads to a telomere-to-telomere genome assembly such as that for HG002 or CHM13. While this works beautifully for data derived from sequencing the reference sample (for example data from HG002 cells), aligning clinical samples back to these telomere-to-telomere references results in a large number of unmapped or multimapping telomeric sequences. Even more disconcerting is the distribution of reads across chromosome arms. When aligning HG002 reads to the HG002 references, approximately 1.08% of the telomeric sequences align to each chromosome arm as expected. However, when aligning uniquely mapping reads from clinical samples to this reference, some chromosome arms contain greater than 10% of all telomeric sequences, more indicative of alignment artifacts than true chromosome arm assignments. These observations have recently been published (https://doi.org/10.1371/journal.pcbi.1013915).

d. Even more interesting would be to see variant content based on chromosome inheritance. In III.4, is there evidence of cross over from the maternally-inherited chromosomes?

A preliminary analysis did not reveal evidence for cross over from the maternally-inherited chromosomes. However, due to the limitations described above, we would rather not include this result in the current study but explore it in more depth prior to publication as part of a follow-up manuscript.

e. How does one interpret the detection of variants above background at the end of the telomere in III.4 in Fig. 6d, which implies these variants have not undergone any turnover?

This is indeed a striking observation that surprised us as well. The fact that mutant repeats are highly abundant in III.4 strongly suggests that there is very limited turnover of telomeric sequences over many cell divisions in an individual who lacks the mutant template TERC. While some reads terminate in variant repeats, others terminate in up to 2000 bp of wild type only repeats. The reads that end in variant repeats likely represent chromosome ends where wild type telomeric repeats added early in life have eroded during subsequent rounds of replication, eventually terminating in a section of repeats that were inherited from the mother. These telomeres either originate from telomerase negative cells or have not yet triggered re-elongation by wildtype telomerase. This explanation has been included in the results [lines 311-315; “However, the distal telomere ends in III.4 do not exhibit GTTTAG repeat levels as low as those observed in II.4. Reads terminating in mutant repeats likely represent chromosome

ends in which wild-type telomeric repeats added early in life have eroded through successive rounds of replication, ultimately terminating in a tract of mutant repeats that was inherited from the mother.”]

f. Also in Fig.6d, the analysis slides only to 4 kb from capture probe. While this distribution is reasonably representative for II.3, it is inadequate for III.4 (based on Fig 6a). It would be worthwhile to extend this into the subtelomere, perhaps scaled as percentage along the telomere instead of by KB.

To address the reviewer’s comment, we have changed the presentation of this data (former Figure 6d, now Fig. 2f). Each telomeric read is now divided into 100 equally-sized fragments and the percent of GTTTAG repeats is calculated and plotted from the subtelomere-to-telomere boundary (left) to the distal end of the telomeres (right). This now illustrates more clearly the addition of wild type repeats at the end of the telomeres of III.4, in contrast to the high mutant repeat content in the distal part of telomeres from II.3. It further shows the similarity in mutant repeat content for II.3 and III.4 at ~50% of the telomere, and the increase in variant repeats close to the TVR region of II.4. Additionally, this plot clearly depicts the spike in mutant GTTTAG repeats in the distal 1% of the telomeres caused by the fill-in synthesis previously described [lines 316-326]

3) Clinical narrative and RTEL1 finding.
a. Lines 309-358 – while the narrative of how the RTEL1 mutation was found could be instructive to clinicians, it is confusing. Especially the subsequent genotyping of III.2, when her blood sample has subsequently been used to provide II.1’s genotypes in Fig. 5c (in conflict with the statement in lines 322-323). I suggest re-writing the clinical narrative, ideally contained in one section of the paper to provide the manifestations, genotypes, and clinical course more succinctly.

We thank the reviewer for this suggestion. All clinical information has now been moved into a single section at the start of the results. We hope that the reviewer will agree that this major restructuring of the manuscript has improved overall clarity for the reader.

b. Except for potentially explaining the severity of clinical manifestations in III.2 and III.3, the RTEL1 mutation finding does not impact the molecular studies on the TERC template and telomere variants, which seem to be the major focus of the manuscript. There could be an argument for describing this finding and potential genotype/phenotype correlations (which are interesting but also variable), transplant courses, etc, in a separate manuscript.

We considered splitting the data into two manuscripts as suggested, but decided against it for the following reasons: While we agree that the RTEL1 finding is a minor portion of our manuscript, it would be impossible to untangle the effects of the RTEL1 mutation from the presence of mutant telomeric repeats in the different

individuals. The co-occurrence of the two mutations thus makes it exceedingly difficult to describe the C50>A hTR mutation in isolation, unless one focusses entirely on the in vitro characterization part. Such a manuscript would confirm the work by Hinchie et al., but would add few new biochemical insights. The strength of the current manuscript is that it expands the published record significantly by describing data from several individuals and leveraging long read sequencing information. We hope that the reviewer agrees that the restructuring of the result section has improved the logical flow and that the RTEL1 result now appears as (small) part of the narrative rather than a confusing distraction.

c. Oligogenic contributions to TBD manifestations have been previously reported and should be discussed/cited (e.g. PMID 26024875).

We thank the reviewer for pointing this out. Oligogenic contributions are now referenced within the discussion on lines 497-499 ["These findings underscore the importance of considering secondary genetic modifiers or oligogenic contributions when evaluating patients with telomere biology disorders[33]."]

Minor comments:

1. Line 105, underlined letters in RTEL1 are incorrect

This has been updated, we thank the reviewer for their attention to detail.

2. Lines 311 and 387, should refer to wild-type TERC, not WT telomerase

The terminology in question has been updated.

3. Line 319, "Clinvar one star" needs more explanation

The definition of ClinVar One Star has been expanded as requested [lines 194-197; "For a variant to achieve the one-star status on ClinVar, the variant must be classified in a way that is consistent or more thorough than current practice guidelines and evidence or rationale for its variant scoring must be provided."].

4. Lines 506-526 re template variant studies: it may be worthwhile including reference/discussion of Jongmans et al (PMID 22341970), showing recurrent somatic genetic reversion in the setting of template domain mutations (del.n.54-57).

We have updated the discussion to include the reference to Jongmans et al.. However, RNA-sequencing evidence shows a similar fraction of mutant and wild type hTERC in this family arguing against somatic reversion at least in the hemopoietic lineage. [lines 526-532; "It is presently unclear whether the mutation arose independently in the two families or whether they share a common ancestor. Hinchie et al. reported an imbalance in mutant and wild-type hTR detected through long non-coding

RNA sequencing[19]. While spontaneous reversions of mutations within *hTERC* have been observed through mitotic recombination in families affected by DC[46], this does not appear to be the case in the family described here as high-coverage total RNA-sequencing evidence showed that the C50>A mutant and wild-type hTR were present in equal proportions."]

5. Lines 559-566, do not contribute meaningfully in this manuscript

This section has been removed from the manuscript.

Reviewer #4 (Remarks to the Author):

I co-reviewed this manuscript with one of the reviewers who provided the listed reports. This is part of the Nature Communications initiative to facilitate training in peer review and to provide appropriate recognition for Early Career Researchers who co-review manuscripts.

We thank reviewer 4 for their contribution to training young scientists in the thorough evaluation of manuscripts.

REVIEWER COMMENTS

Reviewer #1 (Remarks to the Author):

The reviewers have addressed the concerns raised by reviewers 2 and 3 and I support publication of this manuscript.

We sincerely thank the reviewer for dedicating their time to reviewing our manuscript and we appreciate this reviewer's support for publication of this manuscript.

Reviewer #2 (Remarks to the Author):

The authors have made extensive modifications to their manuscript and provided an thoughtful response to my concerns. I support the support the publication of this manuscript.

We thank the reviewer for their positive evaluation of the revised manuscript and for recognizing the improvements made in response to their comments. We greatly appreciate the reviewer's support for publication.

Reviewer #3 (Remarks to the Author):

Deimler, Orioli, and colleagues present a substantially revised manuscript highly responsive to our critiques. The clinical narrative and insights therein are easier to digest and now positioned to amplify the rigorous molecular analyses that follow. It is appreciated that technical elements such as retaining a comparison of Illumina and Nanopore sequencing and identifying artifacts from 3' fill-in during telomere end capture are helpful as the field adopts long-read telomere sequencing.

The additional analysis of III.4 (who has inherited variant telomere repeats but not the TERC mutation) identifying increased G insertion events between two mutant repeats compared to II.3 is an interesting observation and strongly supports a DNA replication "slippage" rather than mutant telomerase mechanism.

We sincerely thank the reviewer for their careful re-evaluation of our revised manuscript. We greatly appreciate the reviewer's recognition of the substantial revision, the improved clinical narrative, and the clearer presentation of molecular analysis that followed. We are also pleased that the reviewer found the inclusion of the Illumina-Nanopore sequencing comparison and the discussion on the 3' fill-in artifacts during end-capture to be valuable contributions to the broader field of long-read telomere sequencing.

As one point of clarification for the new Fig. 6j and text lines 470-479 regarding the observation of decreased mutant repeat tract length: we infer that this is because of the increased DNA replication slippage events between mutant repeats, i.e. as these are prone to harbor extra Gs over time, they do not get "counted" in a consecutive repeat tract. Thus there is a technical underestimation of apparent mutant repeat processivity in an individual like this, because the algorithm detects fewer pure consecutive mutant repeats. Is that correct?

If so, it may be worthwhile clarifying the sentence in lines 475-477 which seems to indicate that there is actually reduced processivity. Then also, in line 477-479, perhaps this should state "another unexpected mechanism by which mutant telomere sequences contribute to accelerated telomere dysfunction" rather than "shortening", if they are indeed not shorter but simply composed of more heterogeneous telomeric sequences (extra Gs) over time. Finally, as a way to further clarify/strengthen this observation, the authors might consider performing an additional analysis where they modify their mutant tract length calling pipeline to tolerate G insertions. Based on their hypothesis that DNA replication leads to G insertions, it would be expected that altering the analysis in this way would eliminate the mutant tract length discrepancy between II.3 and III.4.

The reviewer presents an excellent point. Due to the slippage and incomplete synthesis that was observed early during the initial analysis of the Illumina sequencing data consecutive repeats are not identified by perfect matches. Instead, consecutive mutant repeats were identified by first locating wild-type telomeric repeats and then analyzing the intervening sequence between two wild-type repeats. Mutant telomeric sequence within this fragment (nested between wild type repeats) must account for greater than 95% of the fragment length. However, upon re-investigation this threshold is too strict as sequences like "GGTTTAG" that are flanked by wild type repeats on both sides and arises from the observed slippage behavior, were omitted from the analysis. Under the original criterion, at least 3 consecutive mutant repeats were required before a slippage event would no longer lead to exclusion of an otherwise valid mutant repeat tract. By relaxing this threshold from 95% to 60% (as was previously done in the Illumina data) mutant repeat frequency, we now include GGTTTAG and GGTTTAGGTTTAG and this results in a slight decrease of mutant telomerase processivity from 0.40 to 0.38. It was manually confirmed that this decrease in stringency did not result in the inclusion of erroneous telomeric repeats in the processivity analysis. The text and figures have been updated with the new value where appropriate. Furthermore, the methods section has been updated to include this threshold [lines 832-834, "Mutant repeat tracts were identified by isolating sequences greater than 4 nucleotides between two wild-type repeats and included for analysis if the isolated nucleotides were composed of greater than 60% mutant telomeric repeats"].

Minor comment:
-line 142 – should be RTEL1 in italics

This has been corrected.

Reviewer #4 (Remarks to the Author):

I co-reviewed this manuscript with one of the reviewers who provided the listed reports. This is part of the Nature Communications initiative to facilitate training in peer review and to provide appropriate recognition for Early Career Researchers who co-review manuscripts.
